# Advances in pharmacological interventions for hepatic fibrosis: from pathogenic mechanisms to novel therapeutic targets

**DOI:** 10.1080/07853890.2026.2613502

**Published:** 2026-01-26

**Authors:** Weiqin Zhang, Ting Shao, Caiyan Wang, Shaogui Wang, Lin An

**Affiliations:** State Key Laboratory of Traditional Chinese Medicine Syndrome, International Institute for Traditional Chinese Medicine, School of Pharmaceutical Science, Guangzhou University of Chinese Medicine, Guangzhou, Guangdong, China

**Keywords:** Hepatic fibrosis, Safety and efficacy, Antifibrotic agents, Extracellular matrix, Traditional Chinese Medicine

## Abstract

Hepatic fibrosis (HF) is a pahological consequence of dysregulated wound healing, characterized by excessive deposition of extracellular matrix (ECM) that disrupts liver architecture and function.It is driven by diverse etiologies, including viral, metabolic, cholestatic and toxic insults. Additionally, the pathogenetic mechanisms progressing from initial injury to established fibrosis involve hepatocyte dysfunction, impaired autophagy, oxidative stress, immune modulation, and ultimately the activation of hepatic stellate cells, leading to a sustained imbalance between ECM synthesis and degradation. Consequently, developing effective therapies necessitates strategies targeting both specific etiologies and these core pathological mechanisms.This review systematically examines emerging anti-fibrotic strategies, spanning etiology-specific treatments and inhibitors targeting core pathways (e.g. stellate cell activation, oxidative stress). We evaluate investigational drugs ranging from small molecules and biologics to nano-formulations and highlight multi-targeting natural compounds from Traditional Chinese Medicine (TCM), such as silymarin and resveratrol, which modulate ECM remodeling, inflammation, and metabolism. By integrating preclinical and clinical evidence, this review provides a critical roadmap for developing synergistic therapies that combine modern targeted drugs with natural multi-target agents, addressing a key gap in current research on this integrative approach.

## 1. Introduction

Hepatic fibrosis (HF) is a pathological process characterized by the abnormal proliferation of connective tissue within the liver, triggered by various factors, leading to excessive deposition of extracellular matrix (ECM). While the liver can regenerate after acute damage, chronic injury induces sustained noxious stimuli, resulting in parenchymal distortion. The primary etiologies contributing to liver fibrosis include chronic viral infections, alcoholic liver disease (ALD), and nonalcoholic steatohepatitis (NASH). Additional underlying causes encompass toxin-induced insults (e.g. alcohol, drugs), autoimmune hepatitis, cholestatic disorders, and hereditary metabolic diseases [1].

Cirrhosis, the end-stage condition of HF, is a leading cause of mortality and morbidity worldwide [2]. Chronic liver disease results in over 633,000 cases of cirrhosis annually globally, with a prevalence rate of 0.27% [3]. However, cirrhosis incidence is frequently underestimated due to asymptomatic early stages, explaining the flattened risk curve for incidence compared to mortality. Decompensation occurs in 20–25% of cirrhosis patients, putting 150,000–200,000 patients at risk each year [4]. If left untreated, cirrhosis can progress to hepatocellular carcinoma (HCC), which is one of the leading causes of death in patients with liver cirrhosis [5]. According to the latest global cancer epidemiology report, there were 866,136 new cases of liver cancer, accounting for 4.3% of the global total. The number of new deaths was 758,725, accounting for 7.8% of the global total. It ranks sixth in the world in terms of cancer incidence and third in terms of mortality [6].

Despite this burden, clinically effective anti-fibrotic therapies remain elusive. Liver transplantation is currently the sole curative option for advanced cirrhosis, highlighting the urgent need for pharmacological interventions to halt disease progression. In this review, we consolidate key pharmacological advances in hepatic fibrosis by integrating preclinical and clinical evidence. We seek to bridge disease mechanisms with therapeutic synergies, thereby guiding the development of rational combination regimens and accelerating the translation of integrated treatment strategies into clinical practice.

## 2. Pathologies and mechanisms of HF

HF arises as an immune repair response, characterized by the development of an inflammatory environment and substantial accumulation of ECM. This condition results from a disruption in the balance between the deposition and removal of proteins such as collagen, laminin, elastin, and fibronectin. The causes of liver fibrosis are diverse, including chronic viral hepatitis (hepatitis B, C, and D), alcoholic liver disease, metabolic liver diseases (non-alcoholic fatty liver disease, NAFLD), and cholestatic or autoimmune liver disorders. Persistent injury can lead to fibrotic scarring and eventually lead to liver cirrhosis. In some cases, multiple causes may coexist within an individual. Globally, 57% of cirrhosis cases are attributed to either hepatitis B (30%) or hepatitis C (27%), with alcohol consumption being another significant cause, accounting for approximately 20% of the cases [7].

Epithelial cell injury initiates HF pathogenesis. Damage to hepatocytes in parenchymal liver injury or biliary epithelium in cholestatic diseases is a prerequisite for chronic liver disease. These injuries, caused by inflammation, lead to the activation of hepatic stellate cells (HSCs), which normally store vitamin A and play a pivotal role in the development of HF. Upon activation, HSCs gradually transform into myofibroblasts, a heterogeneous cell population with proliferative, migratory, and fibrotic characteristics. This transformation results in continuous accumulation of α-smooth muscle actin (α-SMA), ECM, and a large amount of type I and type III collagen, leading to scar deposition [8]. Additionally, HSCs secrete transforming growth factor beta 1 (TGF-β1), which triggers a fibrotic response and proliferation of connective tissue. As this cascade of processes continues, fibrous tissue bands separate hepatocyte nodules, eventually replacing the entire liver architecture and resulting in decreased blood flow throughout [9].

Emerging evidence indicates that liver fibrosis is a dynamic and potentially reversible process, suggesting that early detection and intervention can lead to successful treatment and reversal of its progression. Guidelines recommend staging liver fibrosis for all causes of chronic liver diseases to establish prognosis and guide management. When the causative agents or irritants are removed, liver fibrosis and even the progression of cirrhosis may weaken. Consequently, based on the etiology, appropriate drug treatment can be administered, or interventions can target the mechanisms underlying liver fibrosis.

## 3. The treatment of HF targeting different etiologies

**Figure 1.**
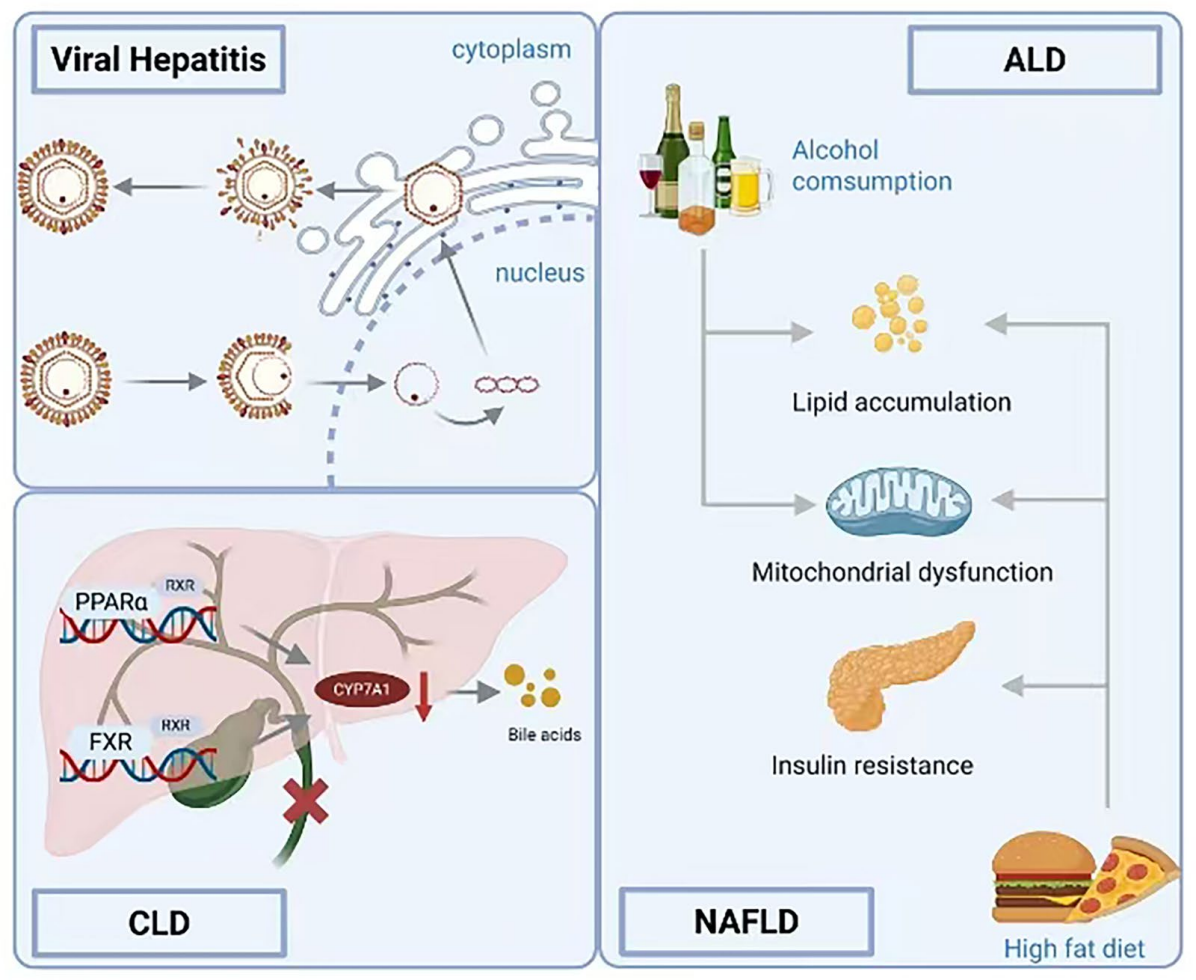
Etiologies of liver fibrosis.

Although cirrhosis-induced liver damage is typically irreversible, timely treatment can halt or slow its progression and reduce the risk of complications. While the fundamental mechanisms underlying fibrosis development are consistent across all chronic liver diseases, each disease has unique etiological features. Therefore, novel anti-fibrotic treatments take these disease-specific factors into account. Liver fibrosis can result from a variety of causes, including viral infections, ethanol consumption, autoimmune conditions, drugs or chemical poisons, genetic disorders, and metabolic diseases. Effectively inhibiting or eliminating the pathogenic factors can reduce ongoing liver damage, thereby promoting the repair and reversal of the fibrotic liver tissue. A promising strategy involves exploring the anti-fibrotic potential of drugs already in clinical use. The following sections review candidate drugs under investigation for HF treatment and the main etiologies of HF are described in Figure 1.

### 3.1. Viral hepatitis

Viral hepatitis, characterized by hepatic inflammation triggered by hepatotropic viral infections (HAV, HBV, HCV, HDV, and HEV), remains a leading cause of liver-related morbidity and mortality worldwide. Chronic HBV and HCV infections collectively account for over 80% of cirrhosis and HCC cases. Notably, HBV alone contributes to 83% of global viral hepatitis-related mortality [10]. According to the 2024 World Health Organization (WHO) report, viral hepatitis-related fatalities surged from 1.1 million in 2019 to 1.3 million in 2022, ranking as the second deadliest communicable disease globally [11].

The most common causes of viral hepatitis are the five unrelated hepatotropic viruses: hepatitis A, B, C, D, and E. HBV infection, caused by a hepatotropic DNA virus, is a major global health issue [10]. Among the deaths attributed to viral hepatitis, 83% are due to HBV infection, which is a common cause of liver disease and a significant contributor to liver fibrosis worldwide. Current treatments for HBV include vaccines, interferons, nucleosides or nucleoside analogs, which reduce new infection rates and the development of liver disease. However, these treatments have not completely prevented the occurrence of severe liver diseases such as cirrhosis and HCC caused by HBV infection [12,13]. Therefore, the development of new antiviral drugs and immune interventions is crucial for optimizing virus suppression programs.

The US Food and Drug Administration (FDA) has approved interferon and nucleoside analogs for the treatment of HBV [14]. Interferons, including ordinary interferon and long-acting interferon, have been translated from basic laboratory research to clinical trials [15]. Despite enhancing antiviral immunity, interferons cause frequent adverse effects (e.g. flu-like symptoms) and yield sustained responses in only 30–40% of patients [16]. Consequently, interferons are not used as first-line drugs for antiviral treatment.

The WHO published guidelines in 2024 for prevention and management of chronic hepatitis B infection [17]. These guidelines state that nucleoside analogs can be used as first-line antiviral drugs to treat chronic hepatitis B infection and are effective in virus suppression. They block reverse transcription in HBV and inhibit its replication, thereby reducing liver necrosis. Although nucleoside analogs reduce liver necrosis and delay complications, they fail to fully prevent cirrhosis or HCC development [12]. Nucleoside analogs effective against HBV include lamivudine (LMV), adefovir (ADV), and entecavir (ETV). These agents inhibit HBV DNA polymerase, blocking viral replication [18]. Their mechanism of action is to inhibit HBV replication. LMV and LdT reduce virus replication by inhibiting the activity of HBV DNA polymerase, preventing the virus from infecting new liver cells until the infected liver cells are cleared or undergo apoptosis. However, nucleoside analogs require long-term treatment to prevent the reactivation of HBV, leading to liver necrosis [19,20]. Additionally, this treatment can lead to drug resistance, with the emergence of drug-resistant hepatitis B mutants during treatment, resulting in treatment failure. HBV covalently closed circular DNA (cccDNA), the genomic template persisting in infected cells, resists elimination by current therapies [21], posing a major challenge in the treatment of HBV.

In recent years, research on mechanisms of HBV replication and pathogenesis has led to the identification of new therapeutic targets and the development of new antiviral drugs. With cccDNA as a new therapeutic target, myrcludex B, ezetimibe, cyclosporin A (CsA), and other drugs have been confirmed to have anti-HBV effects. Myrcludex B and cyclosporin A prevent viral infection by inhibiting the hepatic Na^2+^/taurocholate co-transporting peptide (NTCP), which is central to the entry of HBV into the hepatocytes. Myrcludex B is well tolerated, with no sign of cholestasis in experimental participation, and it does not affect renal function [22]. In a small study with twelve participants, ten experienced 28 adverse events, twelve of which were considered to be due to myrcludex B treatment. All adverse events were transient and minor [23]. CsA is a clinically used immunosuppressant for suppressing the immune response against xenogeneic tissue and is currently being considered as a new anti-HBV drug candidate. However, as CsA inhibits the function of NTCP transporters, it can impair the uptake of sodium-dependent bile acids and may cause significant adverse reactions [24,25]. Researchers have developed derivatives of CsA by modifying the MeBmt sites in its chemical structure and tested their anti-HBV effects in clinical trials. These derivatives include SCY446, SCY806, and SCY450, which can inhibit infection by the A, B, and C genotypes of HBV. Importantly, these compounds are well tolerated and have a wide range of bioactivities [26]. Therefore, CsA derivatives have great potential in developing into genotype-specific anti-HBV drugs, but their antiviral activity, toxicity, *in vivo* pharmacokinetics, and kinetic characteristics require further research and analysis.

GLS4 is a first-in-class capsid assembly modulator that disrupts the capsid assembly of HBV and the reverse transcription into rcDNA of pgRNA to inhibit the replication [27]. The Phase Ia clinical trial of GLS4 showed good tolerability in healthy-adult volunteers. However, due to GLS4 being metabolized mainly by CYP3A4 and the strong first-pass effect, the plasma concentration is low, and the accumulation ratio showed a decreasing tendency when increasing frequency and dose [28]. Therefore, a Phase Ib study was conducted on GLS4 (120 or 240 mg) combined with 100 mg ritonavir, which is used to inhibit CYP3A4 metabolic enzymes. The results showed the therapy with 120 mg GLS4 was well-tolerated and had effective antiviral activity [29]. The Phase IIIa clinical trial of GLS4 is ongoing to further prove its efficiency and safety (Clinical Trials.gov ID: CTR20213273). Additionally, targeting the transcription of cccDNA, there are two classes of drugs: siRNA and antisense oligonucleotides (ASO). Bepirovirsen is an ASO drug that targets all HBV RNAs, including HBV messenger RNA and pregenomic RNA. The results in the Phase IIb trial showed that bepirovirsen can sustain HBsAg and HBV DNA loss in 9–10% of patients for 24 weeks after the end of bepirovirsen treatment, and the phase III clinical trial is underway in multicenter, randomized, and double-blind settings.

Recent research has also found that HCV infection is more likely to progress to liver fibrosis, highlighting the importance of early treatment for HCV. While HCV therapies achieve >95% cure rates, optimizing their integration with anti-fibrotic agents remains an open research question [30,31]. Sofosbuvir is a novel HCV nucleotide analogue NS5B polymerase inhibitor, that can be used as a component of antiviral combination therapy to inhibit the replication of HCV RNA, thereby delaying the process of fibrosis. However, how to combine antiviral therapy with antifibrotic drugs for the treatment of HF still requires more investigations.

### 3.2. Alcoholic liver disease (ALD)

ALD, encompassing steatosis, steatohepatitis, and cirrhosis, results from chronic alcohol misuse, with severity proportional to cumulative intake. Epidemiologic studies indicate that sustained daily consumption of 40–60 g ethanol (≈3–5 standard drinks) for >5 years induces hepatic steatosis in 90% of individuals, while 30% progress to cirrhosis [32]. ALD contributes to 5.1% of global disability-adjusted life years and 5.3% of alcohol-attributable deaths, reflecting unmet therapeutic needs [33].

No FDA-approved pharmacotherapies specifically target ALD. Current management prioritizes alcohol abstinence, nutritional support, and off-label corticosteroid use in severe alcoholic hepatitis. Recent studies implicate dysregulated renin–angiotensin system (RAS) signaling in ALD progression [34]. The use of angiotensin II type 1 receptor blocking agents in patients with compensated ALD has been associated with improvements in fibrosis as assessed by histological and quantitative measurements [35]. In addition, a combination therapy consisting of the ARB candesartan and ursodeoxycholic acid (UDCA) has been shown to improve fibrosis scores in ALD patients [35]. However, conflicting evidence from other trials necessitates further validation of RAS antagonists’ anti-fibrotic efficacy [36]. Therefore, further studies are necessary to confirm the therapeutic potential of RAS antagonists in the treatment of liver fibrosis associated with ALD. Recently, a new study has revealed that HSCs modulate liver zonation and regeneration *via* R-spondin3 (RSPO3) secretion, which activates Wnt/β-catenin signaling in hepatocytes. RSPO3 overexpression ameliorates steatosis and injury in preclinical models, suggesting therapeutic potential [37]. IL-22 emerges as a multi-functional cytokine with hepatoprotective, mitogenic, and anti-fibrotic effects. Its restricted receptor expression minimizes off-target toxicity, positioning it as a promising candidate for ALD therapy [38].

Recent research on the gut–liver axis confirms its potential for treating ALD. Data show over half of individuals with chronic alcohol use have impaired gut barrier function and gut microbiota dysbiosis [39]. Animal studies demonstrate that the change of gut bacteria can alleviate liver injury. For example, supplements like Lactobacillus rhamnosus or VSL#3 improve alcohol-induced liver inflammation and leaky gut [40,41]. Pediococcus pentosaceus reverses gut microbiota dysbiosis and regulates short-chain fatty acids to reduce liver damage in ethanol-induced mice model [42]. Separately, when cytolysin-producing Enterococcus faecalis is targeted by phages, it reduces bacterially mediated liver injury in mice. The intestinal flora plays an important role in maintaining the homeostasis of organisms, and its role in the treatment of ALD has been widely studied. A review summarizing all randomized controlled trials on probiotics for alcohol-related liver injury indicates that probiotics or probiotic combination therapy can effectively improve liver function and reduce inflammation by modulating gut microbiota. Nevertheless, their safety requires further validation [43]. Currently, clinical trials targeting the gut–liver axis in ALD remain limited, highlighting the need for future research to develop effective therapies through this mechanism.

### 3.3. Nonalcoholic fatty liver disease (NAFLD)

NAFLD is a common chronic liver disease globally, characterized by non-inflammatory or mildly inflammatory steatosis and nonalcoholic inflammation or fibrosis steatohepatitis [44]. As the most prevalent liver disorder worldwide, a systematic review indicates that NAFLD affects 30% of adults globally, with an upward trend. Given the liver’s central role in glucose and lipid metabolism, NAFLD is a risk factor for various metabolic diseases. In fact, 90% of patients with type 2 diabetes mellitus (T2DM) develop NAFLD. When T2DM coexists with obesity, 66% of NAFLD patients are expected to have advanced fibrosis [45]. Weight loss through physical activity and caloric restriction is the most effective treatment for NAFLD. However, most people with NAFLD do not perceive their condition as a disease, leading to low motivation for change. If left untreated, NAFLD will progress to liver fibrosis and severe liver disease.

The pathogenesis of NAFLD involves insulin resistance, mitochondrial dysfunction, and concurrent viral infection. Consequently, NAFLD may be managed with vitamin E, pioglitazone, selonsertib, rosuvastatin, liraglutide, simtuzumab, and other drugs [46]. However, the American Association for the Study of Liver Diseases currently does not recommend vitamin E for treating diabetes, NAFLD without liver biopsy, NASH patients with cirrhosis, or occult cirrhosis. Therefore, vitamin E should only be used for NAFLD without diabetes [47]. Thiazolidinediones, widely used in T2DM treatment as insulin sensitizers, have also been applied to NASH. They regulate adiponectin, which enhances lipid and insulin sensitivity. This increases fatty acid synthesis and absorption by adipocytes, reducing their uptake by the liver and muscles [48]. Pioglitazone has been reported to improve histological NASH regarding steatosis, inflammation, hepatocyte swelling, NAFLD activity score (NAS), NASH resolution, and fibrosis improvement. However, the effectiveness of these treatments is not sustainable, with relapse possible after discontinuation. If pioglitazone is used for NASH, indefinite continuation is necessary. Stopping the drug leads to weight gain and no improvement [49]. Moreover, GLP1-receptor agonists (GLP1-RA) and multi-agonists, initially developed for diabetes, are now being tested for NAFLD and NASH treatment. Their mechanisms include glucose-dependent insulin secretion stimulation, glucagon release inhibition, appetite suppression, and delayed gastric emptying, all contributing to improvements in glycemic control, body weight, and blood pressure. Although hepatocytes lack GLP-1 receptors, systemic metabolic improvements (e.g. weight loss, insulin sensitization) mediate hepatic benefits, a recent meta-analysis on NAFLD/MASLD and GLP1-RA showed that liver fat reduction on MRI is linearly related to BMI reduction. The effects on liver fat and inflammation are mediated by favorable metabolic and weight-related actions. Drugs like semaglutide and retatrutide have also demonstrated positive effects on reducing liver fat and improving NAFLD or NASH, showing promise in patients with T2DM and NAFLD or NASH [50].

Statins, as reductase inhibitors, are the most effective lipid-lowering drugs, highly effective in preventing and treating coronary heart disease [51]. Widely used to reduce blood lipids in cardiovascular diseases, studies suggest they may treat NAFLD. A study treated twenty patients with metabolic syndrome (MetS) and biopsy-proven NASH with rosuvastatin monotherapy for one year. Liver biopsy and ultrasonography revealed complete relief of NASH in nineteen cases, with normalized liver enzymes, blood lipids, and blood glucose, and no MetS at the end of the study. This indicates rosuvastatin monotherapy improves biopsy-confirmed NASH and eliminates MetS within 12 months. Statins also improve functional changes and liver histology in NAFLD patients. However, statins can cause elevated serum aspartate and alanine aminotransferase levels, risking liver toxicity [52,53].

On the other hand, endocrine fibroblast growth factor (FGF) analogues have emerged as a promising category because of their ability not only to act directly within the liver but also to promote systemic metabolism. As members of FGF19 subfamily, some FGF21 analogues have been approved to start clinical trials [54,55]. Pegozafermin is a long-acting glycogenated analogue of FGF21 with a longer half-life compared to FGF21. In the liver, FGF21 works by activating adenosine monophosphate-activated protein kinase (AMPK) signaling. This leads to enhanced fatty acid oxidation, reduced de novo lipogenesis, and decreased triglyceride (TG) accumulation. Additionally, it promotes the secretion of TGs in the form of very low-density lipoproteins, which reduces existing fat stores [56]. A phase IIb, double-blind, randomized, placebo-controlled trial investigated the efficacy of pegozafermin in patients with NASH and stage F2 and F3 fibrosis. The results demonstrated significant improvement in fibrosis whereas NASH had no further development, and NASH was resolved without worsening or additional fibrosis [57]. In another randomized controlled trial in patients with SHTG, pegozafermin significantly reduced atherogenic lipoproteins, ApoC3, and liver fat. Although one case of acute pancreatitis occurred in both trials, mild to moderate gastrointestinal side effects were the most common adverse events [58]. FDA has allowed the drug to progress to phase III trials, and the trial has registered in 2024 for F4 NASH. This approval highlights the potential of pegozafermin as a key therapeutic agent for various metabolic disorders. Efruxifermin, also a long-acting FGF21 analogue in clinical trials, significantly reduced liver fat and biomarkers of liver injury and fibrosis, improved glucose and lipid metabolism, and decreased hyperuricemia with tendency for weight loss in a phase IIa study with F1–F3 NASH patients. Notably among these improvements, 11 of 22 patients (50%) with F2 or F3 NASH had stage 2 regression of fibrosis after only 16 weeks of treatment [59]. In the IIb trial for non-alcoholic hepatitis, Efruxifermin was shown to improve liver fibrosis and resolve NASH within 24 weeks in patients with F2 or F3 fibrosis with acceptable tolerance [60]. In addition, its combination with GLP1-RA was demonstrated to significantly improve non-invasive markers of liver injury, fibrosis, glucose and lipid metabolism, while maintaining GLP-1RA-mediated weight loss in a small study, showing great potential for ameliorating liver injury [61].

In March 2024, resmetirom became the first FDA-approved drug for NASH patients with moderate to advanced liver scarring (fibrosis), to be used with diet and exercise. As a thyroid hormone receptor-beta agonist, resmetirom enhances lipid metabolism by promoting thyroid hormone binding to receptors, improving fibrosis and resolving NASH. Its effect is consistent regardless of T2DM presence. Common adverse events (diarrhea, nausea, pruritus) were generally mild, supporting its favorable risk–benefit profile [62].

### 3.4. Cholestatic liver disease (CLD)

CLD occurs when bile flows from the liver to the intestine is impaired. Its etiology encompasses genetic changes, molecular abnormalities induced by drugs, developmental disorders, autoimmune bile duct damage, and structural alterations caused by tumors and gallstones [63]. The term is commonly applied to primary biliary cirrhosis (PBC) and primary sclerosing cholangitis (PSC). PBC has a globally estimated incidence of 1.76 and prevalence of 14.6 per 100,000 individuals, with significant variation across regions. The incidence and prevalence are higher in females and the elderly (60–79 years) [64]. Several FDA-approved drugs have been studied in PBC therapeutic intervention, including UDCA, peroxisome proliferator-activated receptor alpha (PPARα) agonists like seladelpar and elafibranor, and Ocaliva, an farnesoid X receptor (FXR) agonist. These drugs demonstrate certain efficacy in improving PBC, either in combination or as monotherapy.

UDCA, approved by FDA as a first-line treatment, is a secondary bile acid produced by intestinal bacteria. It decreases miR-122 expression, remodels the microbiome, and alters bile acid content, thereby protecting liver function [65]. Numerous randomized controlled trials and meta-analyses have shown that UDCA improves biochemical variables, halts disease progression, and extends liver transplant-free survival, with food efficacy in tolerated patients [66]. However, not all PBC patients respond well to UDCA; approximately 40% have an inadequate reduction in alkaline phosphatase (ALP) or total bilirubin, indicating ineffective biochemical improvement. Additionally, high-dose UDCA (28–30 mg/kg/day) increases serious adverse events, necessitating further research on its appropriate use [67].

Seladelpar and Elafibranor, approved by FDA in 2024 for PBC patients, are PPARδ agonists indicated for use with UDCA in adults with an inadequate response or as monotherapy in that intolerant to UDCA. Their therapeutic mechanisms in PBC are not fully understood, but their pharmacological activity includes inhibiting bile acid synthesis *via* PPARδ activation, a nuclear receptor expressed in most tissues, including the liver. The signaling pathway involves fibroblast growth factor 21 -dependent downregulation of CYP7A1, the key enzyme in bile acid synthesis from cholesterol.

Unlike PBC, PSC primarily affects younger males and is strongly linked to inflammatory bowel disease (IBD). Meta-analysis indicates a 71.1% prevalence of IBD and a 2.16% prevalence of PBC in IBD patients. Some studies suggest increasing incidence and prevalence of PSC [68]. Currently, no officially approved drug therapy exists for PSC. Clinical trials in PSC are challenging due to uncertainties in pathogenesis, the disease’s slow progression, patient heterogeneity, and lack of established endpoints. UDCA at 13–23 mg/kg/day has been considered to elevate ALP/γ-glutamyl transpeptidase in many studies [69]. Recently, Fecal Microbiota Transplantation (FMT) and antibiotics have gained attention for PSC treatment. Ongoing clinical trials show promising prospects for FMT and antibiotics like oral vancomycin in improving PSC biochemical markers, though large clinical studies are needed to confirm their effectiveness [70].

Preclinical studies in a PSC mouse model demonstrated that 24-norursodeoxycholic acid (NorUDCA) exerts potent anti-cholestatic, anti-inflammatory, and anti-fibrotic effects. A Phase II, double-blind, randomized, placebo-controlled trial evaluated NorUDCA in PSC patients for 12 weeks. Compared to placebo, NorUDCA significantly reduced serum ALP, with dose-dependent efficacy up to this threshold. Adverse event rates were comparable to placebo, with nasopharyngitis as the most frequent occurrence [71]. These results, correlating ALP improvement with histological protection in preclinical models, supported NorUDCA’s advancement to global Phase III trials. The drugs for treating liver fibrosis etiology are listed in Table 1.

**Table 1.** Candidate drugs regarding HF etiology.

Compound	Disease	Target	Effect
Interferon	HBV	Cell surface receptor	Getting a higher rate of negative conversion of e antigen
Nucleoside drugs	HBV	Virus	Inhibiting HBV until no detectable HBV DNA negative
Myrcludex B	HBV	Cccdna	Inhibiting the hepatic Na^2+^/taurocholate co-transporting peptide (NTCP)
Cyclosporin A	HBV	Cccdna	Inhibiting the function of NTCP transporters
GLS4	HBV	Capid assembly	Disrupting the capsid assembly of HBV and the reverse transcription into rcdna of pgrna to inhibit the replication
Bepirovirsen	HBV	All HBV rnas	Intervening the transcription of cccdna
Sofebuvir	HCV	Nucleoside NS5B polymerase	Inhibiting the replication of HCV RNA
Semaglutide	NAFLD/NASH	GLP-1 receptor agonist	Reducing liver fat and improving NAFLD or NASH
Retatrutide	NAFLD/NASH	GIP, GLP-1, and glucagon receptors	
Rosuvastatin	NAFLD/NASH	Reductase inhibitor	Lowering the levels of cholesterol in liver and low-density lipoprotein cholesterol and triglycerides in serum
Resmetirom	NASH	Thyroid hormone receptor-beta agonist	Promoting the binding of thyroid hormone to corresponding receptors to enhance lipid metabolism
UDCA	PBC		Decreasing the expression level of mir-122, remodeling the microbiome, and changing the bile acid content
Seladelpar	PBC	PPARδ agonist	Potentially exciting PPARδ to inhibit bile acid synthesis
Elafibranor			
Norudca	PSC		Increasing TGR5 levels to improve biliary protection and healing.

## 4. Therapeutics targeting pathogenesis mechanisms of HF

While eliminating primary etiology is the most effective therapy against HF, targeting pathogenesis mechanisms also helps to alleviate or reverse it. For instance, anti-inflammatory agents may be more beneficial in intermediate disease stages before advanced cirrhosis, whereas matrix-degrading drugs could be particularly useful in more advanced disease. Liver fibrosis results from the liver’s wound-healing response to repeated injury [72]. After acute injury, damaged hepatocytes trigger tissue repair involving inflammation and limited ECM deposition by myofibroblast. Normally, anti-fibrotic mechanisms then counteract fibrotic tissues, leading to myofibroblast inactivation or apoptosis of and scar resolution. However, chronic liver injury causes persistent parenchymal cell damage and disrupts normal tissue repair, resulting in sustained myofibroblast activation and excessive ECM production [73]. HSCs, the main ECM-producing cells in the injured liver [74], are activated by Kupffer cells (KCs) and other immune cells to transdifferentiation from quiescent vitamin A-storing cell to proliferative myofibroblast [75–77]. While studies show HSCs are the main myofibroblast source, other origins include epithelial cells, mesenchymal stromal cells (MSCs), fibrocytes, mesothelial cells, and portal fibroblasts. A complex interplay among different hepatic cell types occurs during fibrogenesis [78,79].

**Figure 2.**
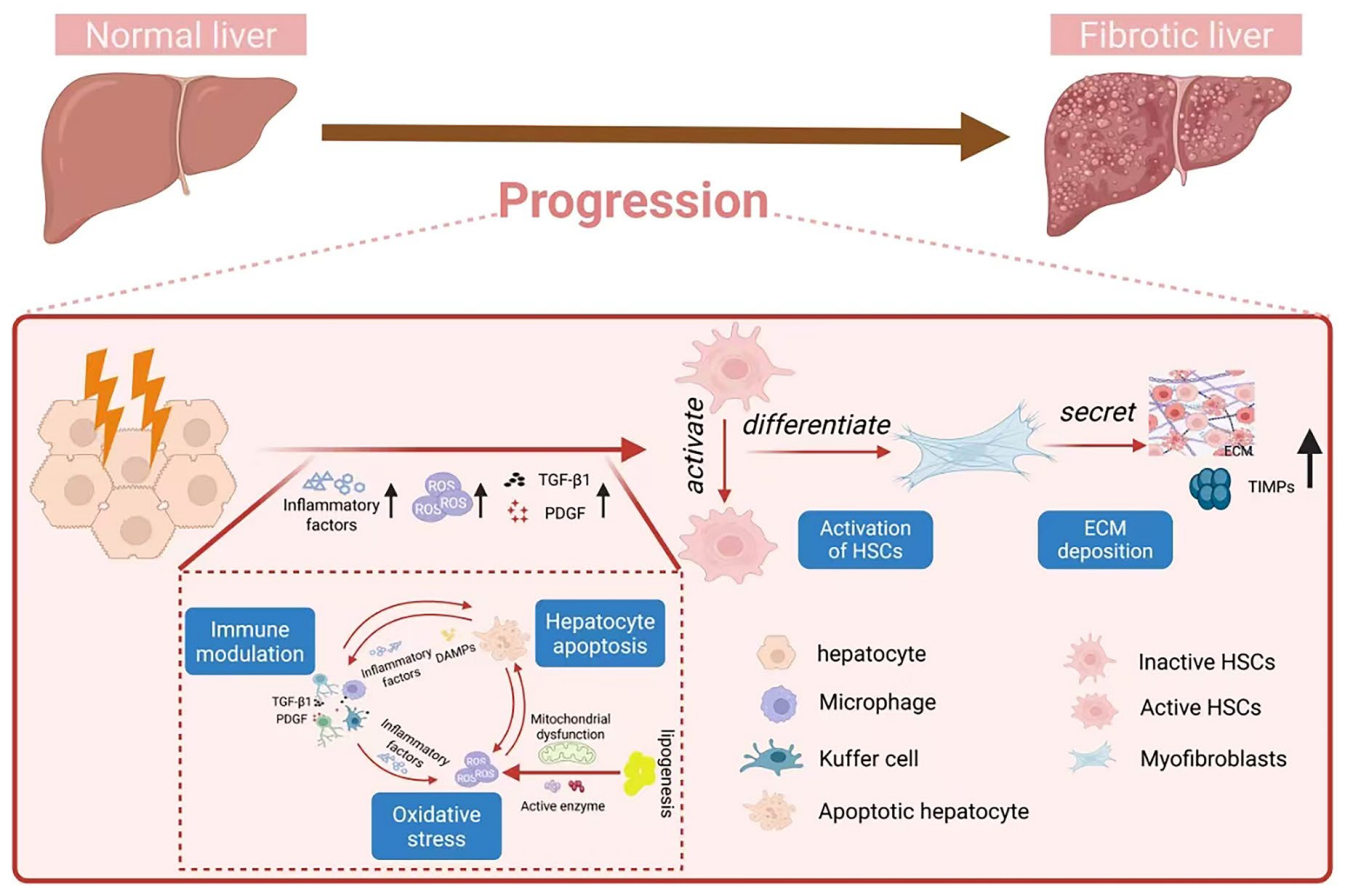
Pathogenesis mechanism of liver fibrosis.

Dead hepatocytes release DAMPs, which activate KCs and HSCs *via* Toll-like receptors (TLRs) [80]. Damaged hepatocytes release reactive oxygen species (ROS) and fibrogenic mediators, inducing inflammatory cell infiltration, while dead hepatocytes release DAMPs that activate surrounding HSCs and KCs. Hepatocyte apoptosis can also promote fibrogenesis through Fas death receptor activation. In response to injury, inflammatory cells like KCs, lymphocytes, leukocytes or neutrophils produce compounds that activate HSCs to secrete collagen [81]. Moreover, liver sinusoidal endothelial cells contribute to HSCs activation by producing fibronectin, TGF-β1, and platelet-derived growth factor (PDGF). Platelets, producing TGF-β1, PDGF and epidermal growth factor (EGF), are also important paracrine stimuli in HSCs activation and fibrogenesis [82,83]. Activated HSCs secrete inflammatory chemokines, express cell adhesion molecules, and modulate the inflammatory response [84]. Persistent HSCs activation disrupts the balance between ECM deposition and dissolution, triggering progressive liver fibrosis [85]. Finally, changes in ECM composition and quantity can directly stimulate fibrogenesis. The pathogenesis mechanisms of HF are summed up in Figure 2. Defining criteria for selecting future antifibrotic treatment candidates is increasingly complex. Several points of attack in developing antifibrotic agents are detailed in subsequent sections and summarized in Table 2.

**Table 2.** Potential molecules about HF mechanisms.

Compound	Target/mechanism	Key pathways/pharmacodynamic effects
VX-166	Pan-caspase inhibitor	Reducing α-SMA expression in HSCs and attenuating fibrosis in NASH models.
Emricasan	Pan-caspase inhibitor	Improving liver function and reducing portal hypertension in cirrhotic models; showing limited effects in clinical trials for NASH.
Glyceraldehyde	Glucose metabolism inhibitor	Inhibiting HSC proliferation by activating caspase-3/9 and upregulating phosphorylated ERK/JNK to induce apoptosis.
Selonsertib	ASK1 inhibitor	Modulating the MAPK pathway to prevent inflammatory cytokine production, downregulate pro-fibrotic genes, and suppress apoptosis and cellular proliferation.
CpG ODN 2088	TLR9 antagonist	Repressing production of fibrotic and inflammatory factors and reducing proliferation and apoptosis markers in experimental fibrosis.
Coenzyme Q10	Mitochondrial function enhancer	Attenuating oxidative stress *via* the JNK/YAP pathway to inhibit apoptosis and ameliorate inflammation and liver fibrosis.
NIM811	Mitochondrial permeability transition pore inhibitor	Inhibiting TGF-β signaling and decreasing fibrosis markers in CCl_4_-induced liver fibrosis.
Setanaxib	NOX1/NOX4 inhibitor	Blocking NOX1/4 activity to reduce ROS production, thereby decreasing liver enzymes and inflammatory markers.
Azathioprine	Purine synthesis inhibitor	Suppressing lymphocyte proliferation and immunoblast formation by inhibiting purine synthesis, thereby exerting immunosuppressive effects.
Cenicriviroc	CCR2/CCR5 dual antagonist	Reducing pro-inflammatory macrophage recruitment and exhibiting anti-fibrotic effects; lowering hs-CRP and fibrinogen in clinical trials.
Belapectin	Galectin-3 inhibitor	Exerting anti-fibrotic effects by inhibiting galectin-3, showing efficacy in preclinical models and a favorable safety profile in early-phase trials.
ICG-001	CBP/β-catenin inhibitor	Disrupting CBP/β-catenin interaction and inhibiting TGF-β-mediated upregulation of α-SMA and collagen I in fibroblasts and HSCs.
PRI-724	CBP/β-catenin inhibitor	Inhibiting HSC activation and collagen production in HCV transgenic mice and CCl_4_∼-induced models.
Rilpivirine	HIV reverse transcriptase inhibitor	Controlling liver cell proliferation and inactivating HSCs *via* modulation of STAT1/STAT3-mediated signaling pathways.
Cabotegravir	HIV integrase inhibitor	Preventing viral DNA integration into the host genome by inhibiting HIV integrase strand transfer activity.
TB001	GLP-1R/GCGR dual agonist	Attenuating ECM accumulation dose-dependently; suppressing HSC activation *via* the TGF-β/Smad pathway and alleviating fibrosis by blocking NF-κB/IκBα and JNK-dependent hepatocyte apoptosis pathways.

### 4.1. Targeting hepatocyte apoptosis in HF

Hepatocyte apoptosis is a crucial trigger and the initial event of inflammation and HSCs activation in liver fibrogenesis across all etiologies [86,87]. Inhibiting hepatocyte apoptosis reduces HSCs activation in animal models of liver fibrosis [88]. For instance, the pan-caspase inhibitor VX-166 reduced α-SMA-positive HSCs and fibrosis in methionine/choline-deficient diet-fed mice [89]. Another pan-caspase inhibitor, Emricasan, showed promise in a carbon tetrachloride (CCl_4_)-based liver fibrosis rat model [90]. Subsequent randomized placebo-controlled clinical trials investigated Emricasan’s effect in NASH patients with F1–F3 fibrosis or cirrhotic patients’ severe portal hypertension [91,92]. Discrepancies between preclinical and clinical outcomes may stem from interspecies variation in apoptosis pathways or compensatory necroptosis activation in human NASH [92]. In cirrhotic NASH patients, Emricasan had a small effect on reducing hepatic venous pressure gradient (HVPG), but no effect in patients with acutely decompensated cirrhosis. A clinical trial of Emricasan in post-transplant HCV-induced fibrosis after SVR is ongoing (NCT02138253).

Restraining HSCs proliferation and promoting their apoptosis are also strategies to improve liver fibrosis. Glyceraldehyde (GA), an inhibitor of glucose metabolism, inhibited cell proliferation in human stellate LX-2 cells by activating caspase-3/9 and upregulating phosphorylated extracellular regulated protein kinases (ERK) and c-Jun N-terminal kinase (JNK) proteins for apoptosis. GA also reduced fibrotic markers in LX-2 cells, demonstrating anti-fibrotic effects [93].

Inhibiting stress signaling pathway is another approach to reduce hepatocyte cell death. Apoptosis signal-regulating kinase (ASK1), part of the MAPK pathways, is involved in hepatic apoptosis, inflammation, and fibrosis [94,95]. The selective ASK1 inhibitor Selonsertib improved fibrosis in a murine NASH model and in a Phase II clinical trial in patients with NASH F2-3 fibrosis [96]. Another Phase II trial showed that combining Selonsertib with Cilofexor or Firsocostat improved steatosis and early-stage liver fibrosis (F1) when combined with Cilofexor [97]. However, Phase III clinical trials in patients with NASH-associated F3 (NCT03053050) and F4 fibrosis (NCT03053063) showed no improvement compared to placebo. These trials confirmed Selonsertib’s positive effects on early-stage NASH, but not advanced liver fibrosis or cirrhosis [98].

Hepatocyte apoptotic bodies can be engulfed and promote TLR9 activation in HSCs and KCs. This results in TGF-β and collagen I production in stellate cells and interleukin-1β generation in KCs. Caspase inhibitors can inhibit death receptor-mediated apoptosis and attenuate liver fibrosis. Similarly, TLR9 inhibitors could prevent stellate cell and Kupffer cell activation, reducing liver fibrosis. Experimental studies suggest blocking TLR9 signaling may be a therapeutic strategy [88]. In a rat trial of CCl_4_-induced liver fibrosis, the TLR9 antagonist CpG ODN 2088 decreased serum ALT and AST, suppressed fibrotic factor production and intrahepatic inflammation, and repressed proliferation and apoptosis markers. These results indicate CpG ODN 2088 could be a promising therapy for liver fibrosis, though clinical trial proof is still needed [99].

### 4.2. Targeting oxidative stress

Oxidative Stress is a major negative regulator in liver fibrosis progression. Normally, the liver balances ROS production and elimination. However, this balance is disrupted under pathological circumstances, leading to excessive ROS accumulation and liver injury. Studies have shown that ROS production is closely related to HSCs activation and ECM deposition during liver fibrosis development [100,101]. Sources of free radicals in liver mainly include mitochondrial metabolism [102], activation of membrane-bound NADPH oxidase (NOX) [103–105], cytoplasmic inducible nitric oxide synthase (iNOS), and microsomal cytochrome P450 [106–108].

Drugs combating mitochondrial dysfunction-induced oxidative stress include Coenzyme Q10 (CoQ10) and NIM811. CoQ10, a component of the mitochondrial electron transport chain, reduced oxidative stress and liver fibrosis in dimethylnitrosamine-induced liver fibrosis mouse model [109]. Clinical studies in NAFLD patients showed CoQ10 decreased liver enzymes, systemic inflammation, and NAFLD severity [110,111]. MitoQ, a mitochondria-targeted CoQ10 derivative, attenuates oxidative stress *via* the JNK/YAP pathway, improving inflammation and liver fibrosis in CCl_4_-induced mice and rats, as well as in human precision-cut liver slices [112]. NIM811, a mitochondrial permeability transition pore inhibitor, decreased fibrosis markers in CCl_4_-induced liver fibrosis in rats [113]. Clinically, NIM811 lowered ALT in HCV genotype 1-infected patients [114].

NOX, which catalyzes oxygen reduction to form superoxide, is closely associated with liver fibrosis. NOX1, NOX2, and NOX4 are the main isoforms linked to HF [115,116]. NOX1 and NOX4 play crucial roles in the HSCs transdifferentiation. NOX4 contributes to fibrosis by activating HSCs and triggering hepatocyte apoptosis, while NOX1 expression in LSEC from high-fat-diet (HFD)-fed mice is associated with enhanced ROS and hepatocellular injury, accelerating NASH progression [112]. NOX4 mRNA and protein levels are elevated in CCl_4_-treated or BDL mice and in NASH patients [117,118]. NOX1 or NOX4 knockout mice exhibited less oxidative stress, inflammation, and fibrosis levels in CCl_4_ treatment compared to wild-type mice [119]. NOX also interacts with other fibrogenesis-related proteins like TLR, angiotensin II, TGF-β, PDGF, and NLRP3. GKT137831 (Setanaxib), a dual NOX1/NOX4 inhibitor, reduced oxidative stress, HSCs activation, inflammation, and fibrogenesis *in vitro* and *in vivo* models of liver fibrosis [119,120]. It also decreased liver enzymes and inflammatory markers in patients with diabetic kidney disease (NCT02010242). Although a Phase II trial in PBC patients did not meet the primary endpoint, secondary endpoints confirmed potential anti-cholestatic and anti-fibrotic effects in PBC. According to the post hoc analysis of a 24-week Phase 2 trial, setanaxib (400 mg BID) may alleviate fatigue and improve emotional well-being in PBC patients. Additionally, patients with clinically significant fatigue saw benefits in social, emotional, and cognitive function [121]. Further studies are needed to explore the clinical usefulness of these oxidative stress inhibitors (NCT03226067).

### 4.3. Targeting immune modulation

Chronic liver inflammation significantly contributes to the development and progression of liver fibrosis, closely linked to hepatocyte necrosis and apoptosis. Research shows that liver fibrosis severity increases with inflammation severity in chronic hepatitis patients [122]. Thus, controlling liver inflammatory responses and regulating abnormal immunity may improve or reverse liver fibrosis. Immunosuppressants are widely used against fibrosis, particularly in autoimmune liver diseases. Many clinical studies indicate that immunosuppressants can improve or reverse autoimmune-induced liver fibrosis. Commonly used immunosuppressants include budesonide, azathioprine, and prednisone. Azathioprine, which inhibit purine synthesis, prevent antigen-sensitive lymphocytes from becoming immunoblasts. However, high doses and long-term use can cause severe bone marrow suppression. Therefore, when treating autoimmune liver disease, azathioprine is combined with other immunosuppressants to ensure efficacy and safety [123].

Beyond immunosuppressants, the dual CCR2/CCR5 inhibitor Cenicriviroc reduces pro-inflammatory macrophage recruitment and exhibits anti-fibrotic effects in liver fibrosis animal models [124,125]. Cenicriviroc was also studied in a Phase II clinical trial for NASH patients (CENTAUR; NCT02217475) [126]. Although Phase II results showed no significant anti-inflammatory effect, patients with advanced fibrosis benefited from Cenicriviroc treatment, which was well-tolerated with only mild adverse events [127,128]. However, a Phase III study on patients with advanced fibrosis and cirrhosis (AURORA; NCT03028740) found no confirmed efficacy, as similar proportion of patients receiving Cenicriviroc or placebo achieved the primary endpoint and complete resolution of steatohepatitis without fibrosis worsening [129].

Stem cell therapy shows promise in alleviating liver inflammation and fibrosis. MSCs secret IL-10, improving liver inflammation and fibrosis, and are major producers of extracellular vesicles (EVs). Bone marrow-derived MSC microvesicles reduce serum ALT, collagen-1α and IL-1β, increase albumin levels, and inactivate the TGF-β/Smad signaling pathway, demonstrating anti-fibrotic, anti-inflammatory, and proangiogenic properties in CCl_4_-induced rat liver fibrosis [130]. Another study shows ES-MSC EVs enhance anti-inflammatory cytokines secretion (e.g. TGF-β1 and IL-10) and reduce IFN-γ levels in TAA-induced chronic rat liver injury, reflecting immunoregulatory activity against liver fibrosis [131]. Despite potential, MSC-EVs require further experimental work before clinical application, including resolving separation, purification, storage, and source tissue [132].

Galectins, carbohydrate-binding proteins secreted by various cells upon liver injury [133], increase in inflammatory, fibrotic, or malignant liver tissue [134,135]. In liver fibrosis, galectins are mainly secreted by activated macrophages [136,137]. Belapectin, a galectin-3 inhibitor, demonstrated anti-fibrotic efficacy in mouse and rat models and was well-tolerated in a Phase I trials [138–140]. However, a phase IIb placebo-controlled study in NASH patients with liver fibrosis showed no significant fibrosis improvement [141]. Despite this, belapectin showed protective effects on hepatocyte ballooning and lower HPVG and varices development in a subgroup of NASH cirrhosis patients, prompting a phase III study in NASH cirrhosis patients without baseline varices.

### 4.4. Targeting hepatic stellate cells activation

In liver fibrosis, HSCs are activated by liver damage, differentiating into proliferative and migratory myofibroblasts that release α-SMA and collagen, causing excessive ECM deposition. Thus, HSCs activation is central to liver fibrosis, and interfering with this process or increasing activated HSCs apoptosis is crucial for treatment. HSCs originate from bone marrow low-density mononuclear cells-derived mesenchymal stem cells. Specific surface receptors on HSCs are targets of biological interventions, attracting much attention. These receptors can serve as carriers for targeted drug delivery, enhance efficacy and reducing adverse reactions [142].

During liver fibrosis development, cytokines like TGF-β, EGF, and hepatocyte growth factor can induce HSCs apoptosis or inhibit activation. Besides directly targeting HSCs receptors, cytokines can intervene in multiple signaling pathways, such as Wnt/β-catenin, TGF-β1/Smad, and PI3K/Akt, effectively inhibiting HSCs proliferation when activated [143]. ICG-001, a small molecule inhibitor disrupting the CREB binding protein (CBP)/β-catenin interaction, inhibits TGF-β mediated upregulation of α-SMA and collagen 1 in mouse fibroblasts and human HSCs [144]. It also inhibits β-catenin/TCF4, promoting portal angiogenesis and suppressing sinusoids capillarization in a bile duct ligation model [145]. In a CCl_4_-induced fibrosis model, ICG-001 attenuates HSCs activation and ECM accumulation, though clinical proof is lacking. PRI-724, another CBP/β-catenin inhibitor, inhibits HSCs activation and collagen production in HCV transgenic mice and shows anti-fibrotic effects in a CCl_4_-induced model [146]. A phase I trial in HCV-associated cirrhosis patients showed dose-dependent histological improvement. A phase I/IIa trial in hepatitis B or C-related cirrhosis patients demonstrated improved liver stiffness, model for end-stage liver disease score, and serum albumin level. However, one of three patients had serious adverse events possibly related to PRI-724, and hepatic fibrosis did not decrease in 12 weeks using PRI-724, warranting further evaluation in cirrhosis patients (NCT03620474).

Rilpivirine (RPV), an antiretroviral drug for HIV infection, controls liver proliferation and inactivates HSCs *via* STAT1/3-mediated pathways, intervening in liver fibrosis development. Clinical data analysis supports this conclusion [147]. Cabotegravir (CAB), another antiretroviral targeting HIV integrase, was approved by FDA in 2021 for combination with RPV in HIV treatment. Cabenuva (CAB/RPV), an intramuscular nano-formulation, is the first FDA-approved long-acting antiretroviral injectable. Its efficacy and safety were confirmed in Phase III trials (FLAIR and ATLAS), showing non-inferiority to oral therapy and equivalent efficacy for maintaining HIV-1 suppression, potentially preventing HIV-related complications. The main side effect was injection site reactions, decreasing over time [148].

### 4.5. Intervention of ECM synthesis and degradation

Liver fibrosis is characterized by increased ECM synthesis and reduced degradation, particularly type I and III collagens. Matrix metalloproteinases (MMPs) and tissue inhibitors of metalloproteinase (TIMPs) are key regulators of ECM balance [149]. MMPs are proteolytic enzymes (e.g. MMP1, MMP2, and MMP9), while TIMPs are natural glycoproteins that inhibit the activation of the MMPs (e.g. TIMP1, TIMP2). Common MMPs inducers include colchicine, allicin, and resveratrol; TIMP1 is a MMP1 specific inhibitor and adenosine receptor agonist.

Small leucine zipper protein (sLZIP), a transcription factor, was found to negatively regulate liver fibrosis. sLZIP knockdown increased PAI-1 transcription and HSCs activation, reducing blood clot lysis and MMP2/9 expression. However, further studies are needed to confirm its role [150]. An imbalance in MMPs and TIMP1 secretion is a primary cause of ECM accumulation in liver fibrosis [8]. A study demonstrated that a recombinant adeno-associated virus harboring siRNA targeting the TIMP-1 gene (rAAV/siRNA-TIMP-1) inhibited macrophage migration in rats by suppressing TIMP-1 in HSCs and affected MCP-1 expression *via* Fli-1 induction [151]. *In vitro* and *in vivo* studies showed that Inhibiting TIMP1 expression blocked the apoptosis-fibrosis link, downregulated fibrosis-related genes (α-SMA, CTGF), and reduced c-Jun expression in liver fibrosis models, highlighting TIMP1 as a potential therapeutic target [152].

Collagen 1 (Col1), the most abundant collagen in fibrotic livers, is a key focus [153]. Specific knockdown of Col1A1 decreases total collagen accumulation [154]. Hsp47, a Col1 chaperone, can be targeted with siRNA to block collagen synthesis. Vitamin A-coupled liposomes containing Hsp47 siRNA, preferentially taken up by HSCs, show significant anti-fibrotic effects in liver fibrosis [155]. BMS 986263, an HSP47 siRNA lipid nanoparticle, demonstrated no toxicity in humans in a clinical trial (NCT02227459).

TB001, a dual glucagon-like peptide-1 receptor/glucagon receptor (GLP-1R/GCGR) agonist, showed dose-dependent attenuation of ECM accumulation in animal models (CCl_4_, anaphthyl-isothiocyanate, bile duct ligation, and Schistosoma japonicum). It reduced HSCs activation *via* TGF-β/Smad pathway suppression and alleviated liver fibrosis by blocking downstream activation of pro-inflammatory nuclear factor κB/NF-κB inhibitor α (NF-κB/IκBα) pathways as well as JNK-dependent induction of hepatocyte apoptosis. These evidences indicate that TB001 is a promising therapeutic candidate for liver fibrosis [156].

## 5. Treatment of liver fibrosis with traditional Chinese medicine

In Traditional Chinese Medicine (TCM), liver fibrosis is taken into the categories of ‘pain’, ‘accumulation’, and ‘symptoms’, characterized by ‘qi’ and ‘yin’ deficiency, blood stasis, blocked meridians, and phlegm coagulation from damp-heat [157]. TCM offers abundant resources for developing anti-inflammatory, anti-infective, and antifibrotic medicine. Many clinical studies report antifibrotic effect of TCM ingredients. Commonly used single herbs include danshen, bupleurum, turmeric, angelica, cordyceps, and glycyrrhiza, which promote blood circulation and remove stasis. Patent medicines like Fuzheng Huayu capsule, Fufang 861, Qianggan capsule, Yigankang, and Fufang Biejia Ruangan tablets enhance liver function in cirrhosis, either as monotherapy or combined with etiology-targeted agents [158–161]. Studied antifibrotic TCM components include alkaloids (tetrandrine, and marine. etc.), organic acids (glycyrrhizic acid, taurine, etc.), flavonoids (danshensu, salvianolic acid B, emodin, naringin, etc.). These components inhibit fibrosis through various mechanisms and have multiple targets, offering advantages over single ingredients. TCMs that were studied based on modern pharmacology or TCM theory mentioned in this paper were listed in Table 3.

**Table 3.** Potential traditional Chinese medicine.

Name	Effect
Fuzheng Huayu capsule	Protecting hepatocyte, promoting apoptosis and degradation of ECM, repressing the activation of HSC and inflammatory response
Fufang Biejia Ruangan tablet	Inhibiting the proliferation of storing-lipids cell and decreasing the synthesis of collagen
Qianggan capsule	Downregulating TGF-β/smad pathway
Silibinin	Inhibiting oxidative stress and participates in NF-κB pathway to reduce liver fibrosis
Licorice	Regulating energy metabolism disorders, inflammatory and immunity
Resveratrol	Repressing the inflammatory and apoptosis, promoting the degradation of ECM
Curcumin	Improving liver fibrosis by inhibiting the epithelial–mesenchymal transition procession and enhancing the autophagy level of hepatocytes in rats, inhibiting hepatic inflammation, steatosis, fibrosis development, and progression in NASH models

Fuzheng Huayu formula (FZHY), effective against liver fibrosis, targets hepatocyte protection, ECM degradation, and HSCs activation inhibition. Results from a clinical trial indicated that FZHY combined with entecavir was observably more effective in advanced stage CHB than entecavir alone. In addition, there was no significantly difference between the combination and entecavir, which mean a good safety [162,163]. Fufang Biejia Ruangan tablet, the first the National Medical Products Administration (NMPA)-approved antifibrotic Chinese medicine preparation, is ready for clinical trials after animal research [164]. Qianggan capsule (QGC) is a Chinese medicine preparation, mainly containing danshen, astragalus, and codonopsis. Numerous animal studies have reported its effectiveness in improving liver fibrosis; even a clinical trial study has proved its effectiveness. A clinical trial probed into the effect of QGC in CHB patients. The outcomes confirms that QGC can effectively decrease the indicators of hepatic fibrosis and patients’ constitutions inspected by TCM [165]. In the treatment of liver fibrosis caused by NAFLD, QGC reduced liver fibrosis-related markers by downregulating the TGF-β1/SMAD signaling pathway, improving the pathological state of liver fibrosis [166]. Factors corresponding to this pathway were tested in patients with liver fibrosis caused by HBV infection. Among them, PDGF-BB/TGFβ1 and TIMP1 activities were significantly reduced, while MMP1 activity was enhanced. After the treatment of QGC, the inflammation of liver tissue, and the degree of fibrosis significantly improved [165]. However, any adverse reactions need to be addressed and studied further.

Silymarin, a flavonoid complex derived from *Silybum marianum*, has been empirically used for decades as a hepatoprotective agent in chronic liver diseases, particularly for toxic-induced injuries and fibrosis progression [167]. Its bioactive constituent silibinin exerts multifactorial effects through antioxidant, anti-inflammatory, and antifibrotic mechanisms. Preclinical studies demonstrate silibinin’s capacity to attenuate HSCs activation, reduce Kupffer cell-mediated inflammation, and mitigate oxidative stress in CCl_4_-induced fibrotic models [168,169]. Notably, it serves as a specific antidote for α-amanitin poisoning by competitively inhibiting hepatocyte toxin uptake [170,171]. A randomized trial revealed silymarin’s ability to modulate gut microbiota and reduce liver stiffness, though limited by small sample size and uncharacterized metabolite profiles [172]. In NAFLD/NASH and HCV cohorts (*n* = 216), a 12-month regimen of silybin-phospholipid-vitamin E complexes improved histologic parameters including hepatocyte ballooning and fibrosis [173]. These advanced formulations address silymarin’s inherent pharmacokinetic challenges—low water solubility (23–47% oral bioavailability) and extensive first-pass metabolism [174,175]—through phosphatidylcholine conjugation and glycosylation strategies [176]. However, large-scale randomized controlled trials are imperative to validate its therapeutic efficacy beyond supportive care applications [167].

Licorice, widely used in China, has detoxification properties. At present, most Chinese patent medicines approved by NMPA contain licorice as the main ingredient. Licorice contains many biologically active ingredients, including glycyrrhizic acid, glycyrrhetinic acid, and glycyrrhizin. These components regulate energy metabolism, immunity, and reduce liver disease through multiple of mechanisms [177–179]. However, the uncertainty of its targets or signaling pathways limits clinical application.

Resveratrol, a polyphenolic phytoalexin with multimodal antioxidant properties, modulates metabolic pathways critical to liver fibrosis through activation of AMP-activated kinase, nuclear factor (erythroid-derived)-like 2 (Nrf2), and nicotinamide adenine dinucleotide NAD+-dependent deacetylase (SIRT1)—key regulators of oxidative stress and cellular energy homeostasis [180–182]. Preclinical studies demonstrate its capacity to ameliorate histopathological features of NASH and fibrosis in rodent models by suppressing TGF-β1/Smad signaling (downregulating Smad2/3/4 while upregulating Smad7) and reducing extracellular matrix deposition [183,184]. Notably, resveratrol attenuates HSCs activation *via* dual mechanisms: inhibition of pro-fibrotic cytokines (TGF-β1, TNF-α, IL-6) and mitigation of endoplasmic reticulum stress-induced apoptosis [185]. A 12-week randomized trial in NAFLD patients revealed significant reductions in hepatic steatosis and serum ALT levels alongside suppressed NF-κB activity yet failed to demonstrate antifibrotic efficacy [186].

Curcumin, a polyphenolic compound derived from *Curcuma longa*, exhibits multimodal therapeutic effects in chronic liver disease through anti-inflammatory, anti-fibrotic, and metabolic regulatory mechanisms [187]. Preclinical studies demonstrate its capacity to reverse CCl_4_-induced hepatic damage by restoring hepatocyte architecture, suppressing fibrous nodule formation, and normalizing serum aminotransferase levels [188]. Mechanistically, curcumin attenuates fibrosis *via* dual pathways: inhibition of EMT through TGF-β/Smad modulation and enhancement of hepatocyte autophagy to mitigate endoplasmic reticulum stress [188]. These effects correlate with reduced hepatic steatosis and fibrosis progression in NASH rodent models [189,190]. Clinical translation faces pharmacokinetic and methodological challenges. While short-term trials (500–1000 mg/d) in NAFLD cohorts (*n* = 87–80) reported reductions in serum transaminases and ultrasonographic steatosis markers [191,192], critical limitations persist in bioavailability constraints, histological evidence gap, and therapeutic ambiguity [193–195].

## 6. Future prospects

TCM has obvious advantages in the treatment of liver fibrosis, but the clinical application of TCM faces systemic limitations largely due to a disconnect between its theoretical foundations and the demands of modern medical standardization and precision. As TCMs involve multiple components acting synergistically on various targets, it makes it difficult to identify active ingredients and determine precise dosages. Additionally, many bioactive compounds in TCM exhibit poor stability, low bioavailability, and potential interactions with other substances. These factors complicate the collection of reliable pharmacokinetic and pharmacodynamic data. Furthermore, the unique nature of TCM poses regulatory difficulties. Defining the scope of regulatory oversight and developing appropriate evaluation methods remain challenging, hindering the standardized application of TCM. To address these limitations, it is essential to respect the principles of TCM while integrating modern scientific approaches. Therefore, to Advance from empirical practice to evidence-based quantification, the pharmacological substances and mechanisms of TCM should be clarified. This will facilitate the development of optimized delivery systems and ultimately overcome existing barriers in TCM administration.

The transition from preclinical research to clinical trials, and ultimately to official market approval and application, faces numerous challenges in drug development and translation. Preclinical studies are optimized for single-target approaches, primarily focusing on direct drug–target interactions while often neglecting the complex microenvironment of human tissues. Key obstacles in clinical translation include low drug bioavailability, limitations in targeted delivery, the complex pathological mechanisms of diseases, and difficulties in identifying critical biomarkers. However, advances in technologies such as organoid culture, exosome-based delivery systems, nanohydrogel research, and multi-omics approaches are steadily addressing these barriers, offering promising pathways to overcome these challenges. Emerging targeted therapies focus on disrupting profibrotic signaling cascades. PDGFRβ antagonists and integrin αvβ6 inhibitors block fibroblast activation, COL1A1-specific siRNA delivered *via* lipid nanoparticles achieves >70% hepatic uptake, preferentially accumulating in KCs and HSCs to suppress collagen synthesis [154,196]. Advanced delivery systems address fibrosis-stage limitations: cationic nanohydrogels enhance siRNA payload retention in fibrotic niches [197], while graphene oxide nanocomposites polarize macrophages toward MMP9-overexpressing phenotypes for ECM degradation [198]. Vitamin A-coupled liposomes enable HSC-selective delivery of Heat shock protein 47 (Hsp47) siRNA, demonstrating collagen knockdown and fibrosis regression across preclinical models [155]. Early-phase trials in NASH patients confirm short-term safety, though histological efficacy remains unverified [195].

Critical translational challenges persist: (1) Limited HSC-targeting efficiency in advanced fibrosis due to altered vascular architecture; (2) Uncharacterized long-term biocompatibility of graphene-based nanomaterials; (3) Discrepancies between preclinical dosing (e.g. 100 mg/kg siRNA in rodents) and clinically achievable regimens [197,198]. Only 12% of antifibrotic candidates advance to Phase III trials, emphasizing the need for humanized liver models and non-invasive biomarkers (e.g. PRO-C3 collagen fragments) to bridge this gap [199]. (4) The pharmacokinetics of various drugs may be altered in the fibrotic pathological environment. Clinical studies on Carvedilol have demonstrated that cirrhotic patients exhibit higher serum drug concentrations compared to normal subjects [200]. In a CCl_4_-induced rat model of liver fibrosis, administration of a single dose of Carvedilol resulted in a significantly increased area under the plasma concentration-time curve. This indicates delayed drug clearance [201]. Therefore, clinical dosage adjustments should be guided by actual pharmacokinetic data. This approach helps minimize the impact of altered drug bioavailability or potential toxicity on therapeutic outcomes.

The management of liver fibrosis remains anchored in etiological control, particularly through antiviral therapies that prevent fibrotic progression and may induce reversal in early stages. Notably, novel HBV therapies targeting cccDNA *via* CRISPR–Cas9 systems and core protein allosteric modulators (CpAMs) show promise in achieving functional cure, even in advanced fibrosis [199,202]. However, therapeutic efficacy diminishes in decompensated cirrhosis due to irreversible architectural distortion, underscoring the need for stage-specific antifibrotic strategies targeting activated HSCs, ductular reaction components, and ECM cross-linking enzymes [196]. Etiological precision must align with microenvironment remodeling. Future pipelines should prioritize: (1) AI-driven multitarget drug design leveraging phytochemical libraries; (2) Longitudinal studies correlating liquid biopsy markers (cfDNA, extracellular vesicles) with fibrosis regression; (3) Adaptive trial designs integrating patient stratification by fibrosis stage and molecular profiling.

Based on the compounds reviewed, the most promising candidates that have progressed to Phase III trials include GLS4 and bepirovirsen for chronic Hepatitis B, both of which represent novel strategies for functionally curing HBV by directly targeting the persistent cccDNA reservoir or its transcriptional activity. In the realm of metabolic dysfunction-associated steatohepatitis (MASH), long-acting FGF21 analogues like pegozafermin and efruxifermin have demonstrated impressive efficacy in resolving fibrosis and steatohepatitis in Phase II studies. Additionally, PPARδ agonists such as seladelpar and elafibranor for primary biliary cholangitis (PBC) highlight the continued potential of nuclear receptor targeting in cholestatic liver disease. The promising outlook for these agents stems from their targeted mechanisms that address key pathogenic drivers-viral persistence, metabolic dysregulation, and bile acid homeostasis-coupled with robust mid-phase clinical data showing significant histological improvement and generally acceptable safety profiles. Their development reflects a strategic shift towards etiology-specific, mechanism-based therapies. Future success will likely depend on further validating these approaches in broader patient populations, optimizing combination regimens, and leveraging predictive biomarkers to identify patients most likely to benefit. This focused, pathway-driven drug development strategy holds considerable promise for delivering new antifibrotic treatments to the clinic in the coming years. Additionally, the gut–liver–brain axis emerges as a multifactorial therapeutic frontier. FXR agonists (e.g. obeticholic acid) and microbiota-directed interventions modulate bile acid homeostasis and neuroinflammation, attenuating fibrosis progression in preclinical NASH models [203,204]. Combinatorial approaches integrating these strategies with traditional medicine derivatives (e.g. curcumin–phospholipid complexes) may synergistically target inflammatory and fibrotic pathways [205].

## Data Availability

No data, models, or code were generated or used during the study.

## Abbreviations

α-SMA: Alpha-smooth muscle actin
ADV: Adefovir
ALD: Alcoholic liver disease
ALP: Alkaline phosphatase
AMPK: Adenosine monophosphate-activated protein kinase
ASK1: Apoptosis signal-regulating kinase
ASO: Antisense oligonucleotides
CAB: Cabotegravir
CAB/RPV: Cabenuva
CBP: The CREB binding protein
cccDNA: Covalently closed circular DNA
CCl_4_: Carbon tetrachloride
CLD: Cholestatic liver disease
Col1: Collagen 1
CoQ10: Coenzyme Q10
CpAMs: Core protein allosteric modulators
CsA: Cyclosporin A
ECM: Extracellular matrix
EGF: Epidermal growth factor
ERK: Extracellular regulated protein kinases
ETV: Entecavir
EVs: Extracellular vesicles
FDA: Food and Drug Administration
FGF: Endocrine fibroblast growth factor
FMT: Fecal microbiota transplantation
FXR: Farnesoid X receptor
FZHY: Fuzheng Huayu formula
GA: Glyceraldehyde
GLP-1R/GCGR: Glucagon-like peptide-1 receptor/glucagon receptor
GLP1-RA: GLP1-receptor agonists
HCC: Hepatocellular carcinoma
HF: Hepatic fibrosis
HFD: High fat diet
HSCs: Hepatic stellate cells
Hsp47: Heat shock protein 47
HVPG: Hepatic venous pressure gradient
IBD: Inflammatory bowel disease
iNOS: Inducible nitric oxide synthase
JNK: C-Jun N-terminal kinase
KCs: Kupffer cells
LMV: Lamivudine
MetS: Metabolic syndrome
MMPs: Matrix metalloproteinases
MSCs: Mesenchymal stromal cells
NADPH: Nicotinamide adenine dinucleotide phosphate
NAFLD: Non-alcoholic fatty liver disease
NAS: NAFLD activity score
NASH: Nonalcoholic steatohepatitis
NF-κB/IκBα: Nuclear factor κB/NF-κB inhibitor α
NorUDCA: 24-norursodeoxycholic acid
NOX: Nicotinamide adenine dinucleotide phosphate oxidase
Nrf2: Nuclear factor (erythroid-derived)-like 2
NTCP: Na^2+^/taurocholate co-transporting peptide
PBC: Primary biliary cirrhosis
PDGF: Platelet-derived growth factor
PPARα: Peroxisome proliferator-activated receptor alpha
PSC: Primary sclerosing cholangitis
QGC: Qianggan capsule
RAS: Renin–angiotensin system
ROS: Reactive oxygen species
RPV: Rilpivirine
RSPO3: R-spondin3
SHTG: Severe hypertriglyceridemia
SLZIP: Small leucine zipper protein
T2DM: Type 2 diabetes mellitus
TCM: Traditional Chinese medicine
TG: Triglyceride
TGF-β1: Transforming growth factor beta 1
TIMPs: Tissue inhibitors of metalloproteinase
TLRs: Toll-like receptors
UDCA: Ursodeoxycholic acid
WHO: World Health Organization

## References

[1] Fan W, Liu T, Chen W, et al. Ecm1 prevents activation of transforming growth factor beta, hepatic stellate cells, and fibrogenesis in mice. Gastroenterology. 2019;157(5):1352–1367 e1313. doi: 10.1053/j.gastro.2019.07.036. 31362006

[2] Vilar-Gomez E, Calzadilla-Bertot L, Wai-Sun Wong V, et al. Fibrosis severity as a determinant of cause-specific mortality in patients with advanced nonalcoholic fatty liver disease: a multi-national cohort study. Gastroenterology. 2018;155(2):443–457 e417. doi: 10.1053/j.gastro.2018.04.034. 29733831

[3] Scaglione S, Kliethermes S, Cao G, et al. The epidemiology of cirrhosis in the united states: a population-based study. J Clin Gastroenterol. 2015;49(8):690–696. doi: 10.1097/MCG.0000000000000208. 25291348

[4] Gustot T, Moreau R. Acute-on-chronic liver failure vs. traditional acute decompensation of cirrhosis. J Hepatol. 2018;69(6):1384–1393. doi: 10.1016/j.jhep.2018.08.024. 30195459

[5] Singal AG, Tiro J, Li X, et al. Hepatocellular carcinoma surveillance among patients with cirrhosis in a population-based integrated health care delivery system. J Clin Gastroenterol. 2017;51(7):650–655. doi: 10.1097/MCG.0000000000000708. 27870642 PMC5436954

[6] Bray F, Laversanne M, Sung H, et al. Global cancer statistics 2022: Globocan estimates of incidence and mortality worldwide for 36 cancers in 185 countries. CA Cancer J Clin. 2024;74(3):229–263. doi: 10.3322/caac.21834. 38572751

[7] Perz JF, Armstrong GL, Farrington LA, et al. The contributions of hepatitis B virus and hepatitis C virus infections to cirrhosis and primary liver cancer worldwide. J Hepatol. 2006;45(4):529–538. doi: 10.1016/j.jhep.2006.05.013. 16879891

[8] Parola M, Pinzani M. Liver fibrosis: pathophysiology, pathogenetic targets and clinical issues. Mol Aspects Med. 2019;65:37–55. doi: 10.1016/j.mam.2018.09.002. 30213667

[9] Vaidya A, Kale VP. Tgf-beta signaling and its role in the regulation of hematopoietic stem cells. Syst Synth Biol. 2015;9(1–2):1–10. doi: 10.1007/s11693-015-9161-2. PMC4427577 25972984

[10] Kan F, Ye L, Yan T, et al. Proteomic and transcriptomic studies of HBV-associated liver fibrosis of an AAV-HBV-infected mouse model. BMC Genomics. 2017;18(1):641. doi: 10.1186/s12864-017-3984-z. 28830339 PMC5568174

[11] World Health O. Global hepatitis report 2024: action for access in low- and middle-income countries. Geneva: World Health Organization; 2024a.

[12] Petersen J, Thompson AJ, Levrero M. Aiming for cure in HBV and HDV infection. J Hepatol. 2016;65(4):835–848. doi: 10.1016/j.jhep.2016.05.043. 27270043

[13] Xia Y, Stadler D, Lucifora J, et al. Interferon-gamma and tumor necrosis factor-alpha produced by T cells reduce the HBV persistence form, CCCDNA, without cytolysis. Gastroenterology. 2016;150(1):194–205. doi: 10.1053/j.gastro.2015.09.026. 26416327

[14] Dienstag JL, Schiff ER, Wright TL, et al. Lamivudine as initial treatment for chronic hepatitis B in the United States. N Engl J Med. 1999;341(17):1256–1263. doi: 10.1056/NEJM199910213411702. 10528035

[15] Lok AS, McMahon BJ. Chronic hepatitis B: update 2009. Hepatology. 2009;50(3):661–662. doi: 10.1002/hep.23190. 19714720

[16] Zoulim F. Hepatitis B virus resistance to antiviral drugs: where are we going? Liver Int. 2011;31 Suppl 1(s1):111–116. doi: 10.1111/j.1478-3231.2010.02399.x. 21205147 PMC3096621

[17] World Health O. Guidelines for the prevention, diagnosis, care and treatment for people with chronic hepatitis b infection (text extract): Executive summary. Infect Dis Immun. 2024b;4(3):103–105. doi: 10.1097/ID9.0000000000000128. 39391287 PMC11462912

[18] Zoulim F, Locarnini S. Hepatitis B virus resistance to nucleos(t)ide analogues. Gastroenterology. 2009;137(5):1593–1608.e2. e1591–1592. doi: 10.1053/j.gastro.2009.08.063. 19737565

[19] Lok ASF, Lai C-L, Leung N, et al. Long-term safety of lamivudine treatment in patients with chronic hepatitis B. Gastroenterology. 2003;125(6):1714–1722. doi: 10.1053/j.gastro.2003.09.033. 14724824

[20] Zoulim F, Perrillo R. Hepatitis B: reflections on the current approach to antiviral therapy. J Hepatol. 2008;48 Suppl 1:S2–S19. doi: 10.1016/j.jhep.2008.01.011. 18304680

[21] Liaw Y-F, Gane E, Leung N, et al. 2-year globe trial results: telbivudine is superior to lamivudine in patients with chronic hepatitis B. Gastroenterology. 2009;136(2):486–495. doi: 10.1053/j.gastro.2008.10.026. 19027013

[22] Huang Q, Zhou B, Cai D, et al. Rapid turnover of hepatitis B virus covalently closed circular DNA indicated by monitoring emergence and reversion of signature-mutation in treated chronic hepatitis B patients. Hepatology. 2021;73(1):41–52. doi: 10.1002/hep.31240. 32189364 PMC7898704

[23] Watashi K, Sluder A, Daito T, et al. Cyclosporin A and its analogs inhibit hepatitis B virus entry into cultured hepatocytes through targeting a membrane transporter, sodium taurocholate cotransporting polypeptide (NTCP). Hepatology. 2014;59(5):1726–1737. doi: 10.1002/hep.26982. 24375637 PMC4265264

[24] Bogomolov P, Alexandrov A, Voronkova N, et al. Treatment of chronic hepatitis D with the entry inhibitor Myrcludex B: first results of a phase IB/IIA study. J Hepatol. 2016;65(3):490–498. doi: 10.1016/j.jhep.2016.04.016. 27132170

[25] Slijepcevic D, Kaufman C, Wichers CGK, et al. Impaired uptake of conjugated bile acids and hepatitis B virus pres1-binding in na(+) -taurocholate cotransporting polypeptide knockout mice. Hepatology. 2015;62(1):207–219. doi: 10.1002/hep.27694. 25641256 PMC4657468

[26] Vaz FM, Paulusma CC, Huidekoper H, et al. Sodium taurocholate cotransporting polypeptide (slc10a1) deficiency: conjugated hypercholanemia without a clear clinical phenotype. Hepatology. 2015;61(1):260–267. doi: 10.1002/hep.27240. 24867799

[27] Berke JM, Dehertogh P, Vergauwen K, et al. Capsid assembly modulators have a dual mechanism of action in primary human hepatocytes infected with hepatitis B virus. Antimicrob Agents Chemother. 2017;61(8): e00560-17. https://www.ncbi.nlm.nih.gov/pubmed/28584155. doi: 10.1128/AAC.00560-17. PMC5527576 28584155

[28] Zhao N, Jia B, Zhao H, et al. A first-in-human trial of gls4, a novel inhibitor of hepatitis B virus capsid assembly, following single- and multiple-ascending-oral-dose studies with or without ritonavir in healthy adult volunteers. Antimicrob Agents Chemother. 2019;64(1): e01686-19. https://www.ncbi.nlm.nih.gov/pubmed/31636065. doi: 10.1128/AAC.01686-19. PMC7187578 31636065

[29] Zhang H, Wang F, Zhu X, et al. Antiviral activity and pharmacokinetics of the hepatitis b virus (HBV) capsid assembly modulator gls4 in patients with chronic HBV infection. Clin Infect Dis. 2021;73(2):175–182. doi: 10.1093/cid/ciaa961. 32649736 PMC8516514

[30] European Association for the Study of the Liver. Electronic address EEE, Clinical Practice Guidelines Panel C, representative EGB, Panel m. 2020. EASL recommendations on treatment of hepatitis C: final update of the series(⋆). J Hepatol. 2020; 73(5):1170–1218. https://www.ncbi.nlm.nih.gov/pubmed/32956768. doi: 10.1016/j.jhep.2020.08.018. 32956768

[31] Ghany MG, Morgan TR, Panel A-I. Hepatitis c guidance 2019 update: American association for the study of liver diseases-infectious diseases society of America recommendations for testing, managing, and treating hepatitis C virus infection. Hepatology. 2020;71(2):686–721. doi: 10.1002/hep.31060. 31816111 PMC9710295

[32] Patel R, Mueller M. Alcohol-associated liver disease. Treasure Island (FL): StatPearls Publishing; 2025 Jan. 31536239

[33] Narro GEC, Díaz LA, Ortega EK, et al. Alcohol-related liver disease: a global perspective. Ann Hepatol. 2024;29(5):101499. doi: 10.1016/j.aohep.2024.101499. 38582247

[34] Biffi S, Cerbino R, Nava G, et al. Correction: equilibrium gels of low-valence DNA nanostars: a colloidal model for strong glass formers. Soft Matter. 2015;11(18):3734–3734. doi: 10.1039/c5sm90069g. 25868490

[35] Kizilates F, Berk H, Seyman D, et al. Plasmodium falciparum malaria and exchange transfusion application. TurkiyeParazitolDerg. 2015;39(2):151–154. doi: 10.5152/tpd.2015.3515. 26081890

[36] Dwyer KM, Burt JD, Bennett T. International vascularised composite allotransplantation activity: implications for Australia. Med J Aust. 2019;210(2):67–68. doi: 10.5694/mja2.12068. 30712306

[37] Sugimoto A, Saito Y, Wang G, et al. Hepatic stellate cells control liver zonation, size and functions via r-spondin 3. Nature. 2025;640(8059):752–761. doi: 10.1038/s41586-025-08677-w. 40074890 PMC12003176

[38] Kong X, Feng D, Mathews S, et al. Hepatoprotective and anti-fibrotic functions of interleukin-22: therapeutic potential for the treatment of alcoholic liver disease. J Gastroenterol Hepatol. 2013;28 Suppl 1(01):56–60. doi: 10.1111/jgh.12032. 23855297 PMC3779467

[39] Leclercq S, Matamoros S, Cani PD, et al. Intestinal permeability, gut–bacterial dysbiosis, and behavioral markers of alcohol-dependence severity. Proc Natl Acad Sci USA. 2014;111(42):E4485–4493. doi: 10.1073/pnas.1415174111. 25288760 PMC4210345

[40] Chang B, Sang L, Wang Y, et al. The protective effect of vsl#3 on intestinal permeability in a rat model of alcoholic intestinal injury. BMC Gastroenterol. 2013;13:151. doi: 10.1186/1471-230X-13-151. 24138544 PMC4016537

[41] Forsyth CB, Farhadi A, Jakate SM, et al. Lactobacillus GG treatment ameliorates alcohol-induced intestinal oxidative stress, gut leakiness, and liver injury in a rat model of alcoholic steatohepatitis. Alcohol. 2009;43(2):163–172. doi: 10.1016/j.alcohol.2008.12.009. 19251117 PMC2675276

[42] Jiang X-W, Li Y-T, Ye J-Z, et al. New strain of pediococcus pentosaceus alleviates ethanol-induced liver injury by modulating the gut microbiota and short-chain fatty acid metabolism. World J Gastroenterol. 2020;26(40):6224–6240. doi: 10.3748/wjg.v26.i40.6224. 33177795 PMC7596634

[43] Zhou X, Zhou S. Safety and efficacy of probiotics in the treatment of ALD. Medicine (Baltimore). 2025;104(32):e43662. doi: 10.1097/MD.0000000000043662. 40797501 PMC12338215

[44] Shimura S, Watashi K, Fukano K, et al. Cyclosporin derivatives inhibit hepatitis B virus entry without interfering with NTCP transporter activity. J Hepatol. 2017;66(4):685–692. doi: 10.1016/j.jhep.2016.11.009. 27890789 PMC7172969

[45] Gutiérrez-Grobe Y, Juárez-Hernández E, Sánchez-Jiménez BA, et al. Less liver fibrosis in metabolically healthy compared with metabolically unhealthy obese patients with non-alcoholic fatty liver disease. Diabetes Metab. 2017;43(4):332–337. doi: 10.1016/j.diabet.2017.02.007. 28318912

[46] Herath HMM, Kodikara I, Weerarathna TP, et al. Prevalence and associations of non-alcoholic fatty liver disease (NAFLD) in Sri Lankan patients with type 2 diabetes: a single center study. Diabetes Metab Syndr. 2019;13(1):246–250. doi: 10.1016/j.dsx.2018.09.002. 30641706

[47] Chalasani N, Younossi Z, Lavine JE, et al. The diagnosis and management of nonalcoholic fatty liver disease: practice guidance from the American association for the study of liver diseases. Hepatology. 2018;67(1):328–357. doi: 10.1002/hep.29367. 28714183

[48] Violi F, Cangemi R. Pioglitazone, vitamin E, or placebo for nonalcoholic steatohepatitis. N Engl J Med. 2010;363(12):1185–1186; author reply 1186. doi: 10.1056/NEJMc1006581. 20857543

[49] Lutchman G, Modi A, Kleiner DE, et al. The effects of discontinuing pioglitazone in patients with nonalcoholic steatohepatitis. Hepatology. 2007;46(2):424–429. doi: 10.1002/hep.21661. 17559148

[50] Ciardullo S, Muraca E, Vergani M, et al. Advancements in pharmacological treatment of nafld/masld: a focus on metabolic and liver-targeted interventions. Gastroenterol Rep (Oxf). 2024;12:goae029. doi: 10.1093/gastro/goae029. 38681750 PMC11052658

[51] Yaghoubi M, Jafari S, Sajedi B, et al. Comparison of fenofibrate and pioglitazone effects on patients with nonalcoholic fatty liver disease. Eur J Gastroenterol Hepatol. 2017;29(12):1385–1388. doi: 10.1097/MEG.0000000000000981. 29023319

[52] Bril F, Portillo Sanchez P, Lomonaco R, et al. Liver safety of statins in prediabetes or t2dm and nonalcoholic steatohepatitis: post hoc analysis of a randomized trial. J Clin Endocrinol Metab. 2017;102(8):2950–2961. doi: 10.1210/jc.2017-00867. 28575232 PMC5546850

[53] Kargiotis K, Athyros VG, Giouleme O, et al. Resolution of non-alcoholic steatohepatitis by rosuvastatin monotherapy in patients with metabolic syndrome. World J Gastroenterol. 2015;21(25):7860–7868. doi: 10.3748/wjg.v21.i25.7860. 26167086 PMC4491973

[54] Dolegowska K, Marchelek-Mysliwiec M, Nowosiad-Magda M, et al. Fgf19 subfamily members: fgf19 and fgf21. J Physiol Biochem. 2019;75(2):229–240. doi: 10.1007/s13105-019-00675-7. 30927227 PMC6611749

[55] Tillman EJ, Rolph T. Fgf21: an emerging therapeutic target for non-alcoholic steatohepatitis and related metabolic diseases. Front Endocrinol (Lausanne). 2020;11:601290. doi: 10.3389/fendo.2020.601290. 33381084 PMC7767990

[56] Lin X, Liu YB, Hu H. Metabolic role of fibroblast growth factor 21 in liver, adipose and nervous system tissues. Biomed Rep. 2017;6(5):495–502. doi: 10.3892/br.2017.890. 28515909 PMC5431415

[57] Loomba R, Sanyal AJ, Kowdley KV, et al. Randomized, controlled trial of the fgf21 analogue pegozafermin in nash. N Engl J Med. 2023;389(11):998–1008. doi: 10.1056/NEJMoa2304286. 37356033 PMC10718287

[58] Bhatt DL, Bays HE, Miller M, et al. The fgf21 analog pegozafermin in severe hypertriglyceridemia: a randomized phase 2 trial. Nat Med. 2023;29(7):1782–1792. doi: 10.1038/s41591-023-02427-z. 37355760 PMC10353930

[59] Harrison SA, Ruane PJ, Freilich BL, et al. Efruxifermin in non-alcoholic steatohepatitis: a randomized, double-blind, placebo-controlled, phase 2a trial. Nat Med. 2021;27(7):1262–1271. doi: 10.1038/s41591-021-01425-3. 34239138

[60] Harrison SA, Frias JP, Neff G, et al. Safety and efficacy of once-weekly efruxifermin versus placebo in non-alcoholic steatohepatitis (harmony): a multicentre, randomised, double-blind, placebo-controlled, phase 2b trial. Lancet Gastroenterol Hepatol. 2023;8(12):1080–1093. doi: 10.1016/S2468-1253(23)00272-8. 37802088

[61] Harrison SA, Frias JP, Lucas KJ, et al. Safety and efficacy of efruxifermin in combination with a glp-1 receptor agonist in patients with nash/mash and type 2 diabetes in a randomized phase 2 study. Clin Gastroenterol Hepatol. 2025;23(1):103–113. doi: 10.1016/j.cgh.2024.02.022. 38447814

[62] Keam SJ. Resmetirom: first approval. Drugs. 2024;84(6):729–735. doi: 10.1007/s40265-024-02045-0. 38771485

[63] Pastori D, Polimeni L, Baratta F, et al. The efficacy and safety of statins for the treatment of non-alcoholic fatty liver disease. Dig Liver Dis. 2015;47(1):4–11. doi: 10.1016/j.dld.2014.07.170. 25224698

[64] Lv T, Chen S, Li M, et al. Regional variation and temporal trend of primary biliary cholangitis epidemiology: a systematic review and meta-analysis. J Gastroenterol Hepatol. 2021;36(6):1423–1434. doi: 10.1111/jgh.15329. 33141955

[65] Cubero FJ, Peng J, Liao L, et al. Inactivation of caspase 8 in liver parenchymal cells confers protection against murine obstructive cholestasis. J Hepatol. 2018;69(6):1326–1334. doi: 10.1016/j.jhep.2018.08.015. 30144553

[66] You H, Ma X, Efe C, et al. Apasl clinical practice guidance: the diagnosis and management of patients with primary biliary cholangitis. Hepatol Int. 2022;16(1):1–23. doi: 10.1007/s12072-021-10276-6. 35119627 PMC8843914

[67] Imam MH, Sinakos E, Gossard AA, et al. High-dose ursodeoxycholic acid increases risk of adverse outcomes in patients with early stage primary sclerosing cholangitis. Aliment Pharmacol Ther. 2011;34(10):1185–1192. doi: 10.1111/j.1365-2036.2011.04863.x. 21957881 PMC3752281

[68] Barberio B, Massimi D, Cazzagon N, et al. Prevalence of primary sclerosing cholangitis in patients with inflammatory bowel disease: a systematic review and meta-analysis. Gastroenterology. 2021;161(6):1865–1877. doi: 10.1053/j.gastro.2021.08.032. 34425093

[69] Bowlus CL, Arrivé L, Bergquist A, et al. AASLD practice guidance on primary sclerosing cholangitis and cholangiocarcinoma. Hepatology. 2023;77(2):659–702. doi: 10.1002/hep.32771. 36083140

[70] Tan N, Lubel J, Kemp W, et al. Current therapeutics in primary sclerosing cholangitis. J Clin Transl Hepatol. 2023;11(5):1267–1281. doi: 10.14218/JCTH.2022.00068S. 37577219 PMC10412694

[71] Fickert P, Hirschfield GM, Denk G, et al. Norursodeoxycholic acid improves cholestasis in primary sclerosing cholangitis. J Hepatol. 2017;67(3):549–558. doi: 10.1016/j.jhep.2017.05.009. 28529147

[72] Friedman SL. Liver fibrosis – from bench to bedside. J Hepatol. 2003;38 Suppl 1:S38–S53. doi: 10.1016/s0168-8278(02)00429-4. 12591185

[73] Zhou WC, Zhang QB, Qiao L. Pathogenesis of liver cirrhosis. World J Gastroenterol. 2014;20(23):7312–7324. doi: 10.3748/wjg.v20.i23.7312. 24966602 PMC4064077

[74] Gabele E, Brenner DA, Rippe RA. Liver fibrosis: signals leading to the amplification of the fibrogenic hepatic stellate cell. Front Biosci. 2003;8:d69–77. doi: 10.2741/887. 12456323

[75] Campana L, Iredale JP. Regression of liver fibrosis. Semin Liver Dis. 2017;37(1):1–10. doi: 10.1055/s-0036-1597816. 28201843

[76] Krenkel O, Tacke F. Liver macrophages in tissue homeostasis and disease. Nat Rev Immunol. 2017;17(5):306–321. doi: 10.1038/nri.2017.11. 28317925

[77] Marra F. Hepatic stellate cells and the regulation of liver inflammation. J Hepatol. 1999;31(6):1120–1130. doi: 10.1016/s0168-8278(99)80327-4. 10604588

[78] Kalluri R, Neilson EG. Epithelial–mesenchymal transition and its implications for fibrosis. J Clin Invest. 2003;112(12):1776–1784. doi: 10.1172/JCI20530. 14679171 PMC297008

[79] Kmiec Z. Cooperation of liver cells in health and disease. Adv Anat Embryol Cell Biol. 2001;161(III–XIII):1–151. doi: 10.1007/978-3-642-56553-3. 11729749

[80] Hernandez C, Huebener P, Pradere J-P, et al. Hmgb1 links chronic liver injury to progenitor responses and hepatocarcinogenesis. J Clin Invest. 2018;128(6):2436–2451. doi: 10.1172/JCI91786. 29558367 PMC5983315

[81] Casini A, Ceni E, Salzano R, et al. Neutrophil-derived superoxide anion induces lipid peroxidation and stimulates collagen synthesis in human hepatic stellate cells: role of nitric oxide. Hepatology. 1997;25(2):361–367. doi: 10.1053/jhep.1997.v25.pm0009021948. 9021948

[82] Holt AP, Stamataki Z, Adams DH. Attenuated liver fibrosis in the absence of B cells. Hepatology. 2006;43(4):868–871. doi: 10.1002/hep.21155. 16557549

[83] Novobrantseva TI, Majeau GR, Amatucci A, et al. Attenuated liver fibrosis in the absence of B cells. J Clin Invest. 2005;115(11):3072–3082. doi: 10.1172/JCI24798. 16276416 PMC1265860

[84] Viñas O. Human hepatic stellate cells show features of antigen-presenting cells and stimulate lymphocyte proliferation. Hepatology. 2003;38(4):919–929. doi: 10.1053/jhep.2003.50392. 14512879

[85] Mederacke I, Hsu CC, Troeger JS, et al. Fate tracing reveals hepatic stellate cells as dominant contributors to liver fibrosis independent of its aetiology. Nat Commun. 2013;4(1):2823. doi: 10.1038/ncomms3823. 24264436 PMC4059406

[86] Cho G-W, Shin SM, Kim HK, et al. Hccr-1, a novel oncogene, encodes a mitochondrial outer membrane protein and suppresses the uvc-induced apoptosis. BMC Cell Biol. 2007;8(1):50. doi: 10.1186/1471-2121-8-50. 18045496 PMC2222240

[87] Sadagopal S, Lorey SL, Barnett L, et al. Enhanced pd-1 expression by t cells in cerebrospinal fluid does not reflect functional exhaustion during chronic human immunodeficiency virus type 1 infection. J Virol. 2010;84(1):131–140. doi: 10.1128/JVI.01181-09. 19828602 PMC2798447

[88] Watanabe A, Hashmi A, Gomes DA, et al. Apoptotic hepatocyte DNA inhibits hepatic stellate cell chemotaxis via toll-like receptor 9. Hepatology. 2007;46(5):1509–1518. doi: 10.1002/hep.21867. 17705260

[89] Witek RP, Stone WC, Karaca FG, et al. Pan-caspase inhibitor vx-166 reduces fibrosis in an animal model of nonalcoholic steatohepatitis. Hepatology. 2009;50(5):1421–1430. doi: 10.1002/hep.23167. 19676126

[90] Gracia-Sancho J, Manicardi N, Ortega-Ribera M, et al. Emricasan ameliorates portal hypertension and liver fibrosis in cirrhotic rats through a hepatocyte-mediated paracrine mechanism. Hepatol Commun. 2019;3(7):987–1000. doi: 10.1002/hep4.1360. 31304452 PMC6601324

[91] Garcia-Tsao G, Bosch J, Kayali Z, et al. Randomized placebo-controlled trial of emricasan for non-alcoholic steatohepatitis-related cirrhosis with severe portal hypertension. J Hepatol. 2020;72(5):885–895. doi: 10.1016/j.jhep.2019.12.010. 31870950

[92] Harrison SA, Goodman Z, Jabbar A, et al. A randomized, placebo-controlled trial of emricasan in patients with NASH and f1–f3 fibrosis. J Hepatol. 2020a;72(5):816–827. doi: 10.1016/j.jhep.2019.11.024. 31887369

[93] Samsuzzaman M, Kim SY. Anti-fibrotic effects of dl-glyceraldehyde in hepatic stellate cells via activation of erk-jnk-caspase-3 signaling axis. Biomol Ther (Seoul). 2023;31(4):425–433. doi: 10.4062/biomolther.2022.131. 37035877 PMC10315341

[94] Wang P-X, Ji Y-X, Zhang X-J, et al. Targeting casp8 and fadd-like apoptosis regulator ameliorates nonalcoholic steatohepatitis in mice and nonhuman primates. Nat Med. 2017;23(4):439–449. doi: 10.1038/nm.4290. 28218919

[95] Yamamoto E, Dong Y-F, Kataoka K, et al. Olmesartan prevents cardiovascular injury and hepatic steatosis in obesity and diabetes, accompanied by apoptosis signal regulating kinase-1 inhibition. Hypertension. 2008;52(3):573–580. doi: 10.1161/HYPERTENSIONAHA.108.112292. 18678790

[96] Loomba R, Lawitz E, Mantry PS, et al. The ask1 inhibitor selonsertib in patients with nonalcoholic steatohepatitis: a randomized, phase 2 trial. Hepatology. 2018;67(2):549–559. doi: 10.1002/hep.29514. 28892558 PMC5814892

[97] Loomba R, Noureddin M, Kowdley KV, et al. Combination therapies including cilofexor and firsocostat for bridging fibrosis and cirrhosis attributable to NASH. Hepatology. 2021;73(2):625–643. doi: 10.1002/hep.31622. 33169409

[98] Harrison SA, Wong VW-S, Okanoue T, et al. Selonsertib for patients with bridging fibrosis or compensated cirrhosis due to NASH: results from randomized phase III stellar trials. J Hepatol. 2020b;73(1):26–39. doi: 10.1016/j.jhep.2020.02.027. 32147362

[99] Alzahrani B, A M Alameen A, Tantawy A. Therapeutic impact of odn2088 to block tlr9 activity in induced liver fibrosis mice. Pak J Biol Sci. 2021;24(1):122–131. doi: 10.3923/pjbs.2021.122.131. 33683038

[100] Jia Y, Wang F, Guo Q, et al. Curcumol induces ripk1/ripk3 complex-dependent necroptosis via jnk1/2-ros signaling in hepatic stellate cells. Redox Biol. 2018;19:375–387. doi: 10.1016/j.redox.2018.09.007. 30237126 PMC6142373

[101] Kong M, Chen X, Lv F, et al. Serum response factor (SRF) promotes ROS generation and hepatic stellate cell activation by epigenetically stimulating ncf1/2 transcription. Redox Biol. 2019;26:101302. doi: 10.1016/j.redox.2019.101302. 31442911 PMC6831835

[102] Zorov DB, Juhaszova M, Sollott SJ. Mitochondrial reactive oxygen species (ROS) and ROS-induced ROS release. Physiol Rev. 2014;94(3):909–950. doi: 10.1152/physrev.00026.2013. 24987008 PMC4101632

[103] Cordero-Herrera I, Kozyra M, Zhuge Z, et al. Amp-activated protein kinase activation and NADPH oxidase inhibition by inorganic nitrate and nitrite prevent liver steatosis. Proc Natl Acad Sci USA. 2019;116(1):217–226. doi: 10.1073/pnas.1809406115. 30559212 PMC6320503

[104] Crosas-Molist E, Fabregat I. Role of NADPH oxidases in the redox biology of liver fibrosis. Redox Biol. 2015;6:106–111. doi: 10.1016/j.redox.2015.07.005. 26204504 PMC4804101

[105] Kim SY, Jeong J-M, Kim SJ, et al. Pro-inflammatory hepatic macrophages generate ROS through NADPH oxidase 2 via endocytosis of monomeric TLR4–MD2 complex. Nat Commun. 2017;8(1):2247. doi: 10.1038/s41467-017-02325-2. 29269727 PMC5740170

[106] Arauz J, Ramos-Tovar E, Muriel P. Redox state and methods to evaluate oxidative stress in liver damage: from bench to bedside. Ann Hepatol. 2016;15(2):160–173. doi: 10.5604/16652681.1193701. 26845593

[107] Iwakiri Y, Kim MY. Nitric oxide in liver diseases. Trends Pharmacol Sci. 2015;36(8):524–536. doi: 10.1016/j.tips.2015.05.001. 26027855 PMC4532625

[108] Xu J, Ma H-Y, Liang S, et al. The role of human cytochrome p450 2e1 in liver inflammation and fibrosis. Hepatol Commun. 2017;1(10):1043–1057. doi: 10.1002/hep4.1115. 29404441 PMC5721400

[109] Choi H-K, Pokharel YR, Lim SC, et al. Inhibition of liver fibrosis by solubilized coenzyme q10: role of nrf2 activation in inhibiting transforming growth factor-beta1 expression. Toxicol Appl Pharmacol. 2009;240(3):377–384. doi: 10.1016/j.taap.2009.07.030. 19647758

[110] Farhangi MA, Alipour B, Jafarvand E, et al. Oral coenzyme q10 supplementation in patients with nonalcoholic fatty liver disease: effects on serum vaspin, chemerin, pentraxin 3, insulin resistance and oxidative stress. Arch Med Res. 2014;45(7):589–595. doi: 10.1016/j.arcmed.2014.11.001. 25450583

[111] Farsi F, Mohammadshahi M, Alavinejad P, et al. Functions of coenzyme q10 supplementation on liver enzymes, markers of systemic inflammation, and adipokines in patients affected by nonalcoholic fatty liver disease: a double-blind, placebo-controlled, randomized clinical trial. J Am Coll Nutr. 2016;35(4):346–353. doi: 10.1080/07315724.2015.1021057. 26156412

[112] Blas-García A, Apostolova N. Novel therapeutic approaches to liver fibrosis based on targeting oxidative stress. Antioxidants (Basel). 2023;12(8):1567. doi: 10.3390/antiox12081567. 37627562 PMC10451738

[113] Chen J, Liu D-G, Wang H, et al. Nim811 downregulates transforming growth factor‑beta signal transduction *in vivo* and *in vitro*. Mol Med Rep. 2016;13(1):522–528. doi: 10.3892/mmr.2015.4572. 26573209

[114] Lawitz E, Godofsky E, Rouzier R, et al. Safety, pharmacokinetics, and antiviral activity of the cyclophilin inhibitor nim811 alone or in combination with pegylated interferon in HCV-infected patients receiving 14 days of therapy. Antiviral Res. 2011;89(3):238–245. doi: 10.1016/j.antiviral.2011.01.003. 21255610

[115] De Minicis S, Seki E, Paik Y-H, et al. Role and cellular source of nicotinamide adenine dinucleotide phosphate oxidase in hepatic fibrosis. Hepatology. 2010;52(4):1420–1430. doi: 10.1002/hep.23804. 20690191 PMC2947612

[116] Liang S, Kisseleva T, Brenner DA. The role of nadph oxidases (noxs) in liver fibrosis and the activation of myofibroblasts. Front Physiol. 2016;7:17. doi: 10.3389/fphys.2016.00017. 26869935 PMC4735448

[117] Bettaieb A, Jiang JX, Sasaki Y, et al. Hepatocyte nicotinamide adenine dinucleotide phosphate reduced oxidase 4 regulates stress signaling, fibrosis, and insulin sensitivity during development of steatohepatitis in mice. Gastroenterology. 2015;149(2):468–480 e410. doi: 10.1053/j.gastro.2015.04.009. 25888330 PMC4516583

[118] Cai S-M, Yang R-Q, Li Y, et al. Angiotensin-(1–7) improves liver fibrosis by regulating the nlrp3 inflammasome via redox balance modulation. Antioxid Redox Signal. 2016;24(14):795–812. doi: 10.1089/ars.2015.6498. 26728324

[119] Lan T, Kisseleva T, Brenner DA. Deficiency of nox1 or nox4 prevents liver inflammation and fibrosis in mice through inhibition of hepatic stellate cell activation. PLoS One. 2015;10(7):e0129743. doi: 10.1371/journal.pone.0129743. 26222337 PMC4519306

[120] Aoyama T, Paik Y-H, Watanabe S, et al. Nicotinamide adenine dinucleotide phosphate oxidase in experimental liver fibrosis: gkt137831 as a novel potential therapeutic agent. Hepatology. 2012;56(6):2316–2327. doi: 10.1002/hep.25938. 22806357 PMC3493679

[121] Jones D, Carbone M, Invernizzi P, et al. Impact of setanaxib on quality of life outcomes in primary biliary cholangitis in a phase 2 randomized controlled trial. Hepatol Commun. 2023;7(3):e0057–e0057. doi: 10.1097/HC9.0000000000000057. 36809195 PMC9949832

[122] Widjaja AA, Singh BK, Adami E, et al. Inhibiting interleukin 11 signaling reduces hepatocyte death and liver fibrosis, inflammation, and steatosis in mouse models of nonalcoholic steatohepatitis. Gastroenterology. 2019;157(3):777–792 e714. doi: 10.1053/j.gastro.2019.05.002. 31078624

[123] Matsuo K, Sasaki E, Higuchi S, et al. Involvement of oxidative stress and immune- and inflammation-related factors in azathioprine-induced liver injury. Toxicol Lett. 2014;224(2):215–224. doi: 10.1016/j.toxlet.2013.10.025. 24184165

[124] Lefebvre E, Moyle G, Reshef R, et al. Antifibrotic effects of the dual ccr2/ccr5 antagonist cenicriviroc in animal models of liver and kidney fibrosis. PLoS One. 2016b;11(6):e0158156. doi: 10.1371/journal.pone.0158156. 27347680 PMC4922569

[125] Mossanen JC, Krenkel O, Ergen C, et al. Chemokine (c–c motif) receptor 2-positive monocytes aggravate the early phase of acetaminophen-induced acute liver injury. Hepatology. 2016;64(5):1667–1682. doi: 10.1002/hep.28682. 27302828

[126] Friedman S, Sanyal A, Goodman Z, et al. Efficacy and safety study of cenicriviroc for the treatment of non-alcoholic steatohepatitis in adult subjects with liver fibrosis: centaur phase 2b study design. Contemp Clin Trials. 2016;47:356–365. doi: 10.1016/j.cct.2016.02.012. 26944023

[127] Friedman SL, Ratziu V, Harrison SA, et al. A randomized, placebo-controlled trial of cenicriviroc for treatment of nonalcoholic steatohepatitis with fibrosis. Hepatology. 2018;67(5):1754–1767. doi: 10.1002/hep.29477. 28833331 PMC5947654

[128] Lefebvre E, Gottwald M, Lasseter K, et al. Pharmacokinetics, safety, and ccr2/ccr5 antagonist activity of cenicriviroc in participants with mild or moderate hepatic impairment. Clin Transl Sci. 2016a;9(3):139–148. doi: 10.1111/cts.12397. 27169903 PMC5351328

[129] Anstee QM, Neuschwander-Tetri BA, Wai-Sun Wong V, et al. Cenicriviroc lacked efficacy to treat liver fibrosis in nonalcoholic steatohepatitis: Aurora phase III randomized study. Clin Gastroenterol Hepatol. 2024;22(1):124–134 e121. doi: 10.1016/j.cgh.2023.04.003. 37061109

[130] Sabry D, Mohamed A, Monir M, et al. The effect of mesenchymal stem cells derived microvesicles on the treatment of experimental ccl4 induced liver fibrosis in rats. Int J Stem Cells. 2019;12(3):400–409. doi: 10.15283/ijsc18143. 31474025 PMC6881047

[131] Mardpour S, Hassani S-N, Mardpour S, et al. Extracellular vesicles derived from human embryonic stem cell-MSCS ameliorate cirrhosis in thioacetamide-induced chronic liver injury. J Cell Physiol. 2018;233(12):9330–9344. doi: 10.1002/jcp.26413. 29266258

[132] Zhu M, Hua T, Ouyang T, et al. Applications of mesenchymal stem cells in liver fibrosis: novel strategies, mechanisms, and clinical practice. Stem Cells Int. 2021;2021:6546780–6546717. doi: 10.1155/2021/6546780. 34434239 PMC8380491

[133] Barondes SH, Castronovo V, Cooper DN, et al. Galectins: a family of animal beta-galactoside-binding lectins. Cell. 1994;76(4):597–598. doi: 10.1016/0092-8674(94)90498-7. 8124704

[134] Bacigalupo ML, Manzi M, Rabinovich GA, et al. Hierarchical and selective roles of galectins in hepatocarcinogenesis, liver fibrosis and inflammation of hepatocellular carcinoma. World J Gastroenterol. 2013;19(47):8831–8849. doi: 10.3748/wjg.v19.i47.8831. 24379606 PMC3870534

[135] Matter MS, Marquardt JU, Andersen JB, et al. Oncogenic driver genes and the inflammatory microenvironment dictate liver tumor phenotype. Hepatology. 2016;63(6):1888–1899. doi: 10.1002/hep.28487. 26844528 PMC4874846

[136] Sano H, Hsu DK, Yu L, et al. Human galectin-3 is a novel chemoattractant for monocytes and macrophages. J Immunol. 2000;165(4):2156–2164. doi: 10.4049/jimmunol.165.4.2156. 10925302

[137] Yang RY, Hsu DK, Liu FT. Expression of galectin-3 modulates t-cell growth and apoptosis. Proc Natl Acad Sci USA. 1996;93(13):6737–6742. doi: 10.1073/pnas.93.13.6737. 8692888 PMC39096

[138] Harrison SA, Marri SR, Chalasani N, et al. Randomised clinical study: gr-md-02, a galectin-3 inhibitor, vs. placebo in patients having non-alcoholic steatohepatitis with advanced fibrosis. Aliment Pharmacol Ther. 2016;44(11–12):1183–1198. doi: 10.1111/apt.13816. 27778367

[139] Traber PG, Chou H, Zomer E, et al. Regression of fibrosis and reversal of cirrhosis in rats by galectin inhibitors in thioacetamide-induced liver disease. PLoS One. 2013;8(10):e75361. doi: 10.1371/journal.pone.0075361. 24130706 PMC3793988

[140] Traber PG, Zomer E. Therapy of experimental nash and fibrosis with galectin inhibitors. PLoS One. 2013;8(12):e83481. doi: 10.1371/journal.pone.0083481. 24367597 PMC3867460

[141] Chalasani N, Abdelmalek MF, Garcia-Tsao G, et al. Effects of belapectin, an inhibitor of galectin-3, in patients with nonalcoholic steatohepatitis with cirrhosis and portal hypertension. Gastroenterology. 2020;158(5):1334–1345 e1335. doi: 10.1053/j.gastro.2019.11.296. 31812510

[142] Elrick LJ, Leel V, Blaylock MG, et al. Generation of a monoclonal human single chain antibody fragment to hepatic stellate cells: a potential mechanism for targeting liver anti-fibrotic therapeutics. J Hepatol. 2005;42(6):888–896. doi: 10.1016/j.jhep.2005.01.028. 15885360

[143] Wu N, Meng F, Zhou T, et al. The secretin/secretin receptor axis modulates ductular reaction and liver fibrosis through changes in transforming growth factor-beta1-mediated biliary senescence. Am J Pathol. 2018;188(10):2264–2280. doi: 10.1016/j.ajpath.2018.06.015. 30036520 PMC6168967

[144] Emami KH, Nguyen C, Ma H, et al. A small molecule inhibitor of beta-catenin/creb-binding protein transcription [corrected]. Proc Natl Acad Sci USA. 2004;101(34):12682–12687. doi: 10.1073/pnas.0404875101. 15314234 PMC515116

[145] Gao Y, Fan S, Zhao P, et al. Beta-catenin/tcf4 inhibitors icg-001 and lf3 alleviate bdl-induced liver fibrosis by suppressing lect2 signaling. Chem Biol Interact. 2023;371:110350. doi: 10.1016/j.cbi.2023.110350. 36639009

[146] Tokunaga Y, Osawa Y, Ohtsuki T, et al. Selective inhibitor of wnt/beta-catenin/cbp signaling ameliorates hepatitis c virus-induced liver fibrosis in mouse model. Sci Rep. 2017;7(1):325. doi: 10.1038/s41598-017-00282-w. 28336942 PMC5427997

[147] Martí-Rodrigo A, Alegre F, Moragrega ÁB, et al. Rilpivirine attenuates liver fibrosis through selective stat1-mediated apoptosis in hepatic stellate cells. Gut. 2020;69(5):920–932. doi: 10.1136/gutjnl-2019-318372. 31530714

[148] Taki E, Soleimani F, Asadi A, et al. Cabotegravir/rilpivirine: the last FDA-approved drug to treat HIV. Expert Rev Anti Infect Ther. 2022;20(8):1135–1147. doi: 10.1080/14787210.2022.2081153. 35596583

[149] Russo I, Cavalera M, Huang S, et al. Protective effects of activated myofibroblasts in the pressure-overloaded myocardium are mediated through smad-dependent activation of a matrix-preserving program. Circ Res. 2019;124(8):1214–1227. doi: 10.1161/CIRCRESAHA.118.314438. 30686120 PMC6459716

[150] Kang H, Kim S, Park S, et al. Small leucine zipper protein negatively regulates liver fibrosis by suppressing the expression of plasminogen activator inhibitor-1. Exp Cell Res. 2024;442(2):114258. doi: 10.1016/j.yexcr.2024.114258. 39293522

[151] Huang X, Wang X, Wang Y, et al. Timp-1 promotes expression of mcp-1 and macrophage migration by inducing fli-1 in experimental liver fibrosis. J Clin Transl Hepatol. 2024;12(7):634–645. doi: 10.14218/JCTH.2023.00514. 38993513 PMC11233975

[152] Wang K, Lin B, Brems JJ, et al. Hepatic apoptosis can modulate liver fibrosis through timp1 pathway. Apoptosis. 2013;18(5):566–577. doi: 10.1007/s10495-013-0827-5. 23456624

[153] Karsdal MA, Nielsen SH, Leeming DJ, et al. The good and the bad collagens of fibrosis – their role in signaling and organ function. Adv Drug Deliv Rev. 2017;121:43–56. doi: 10.1016/j.addr.2017.07.014. 28736303

[154] Jiménez Calvente C, Sehgal A, Popov Y, et al. Specific hepatic delivery of procollagen alpha1(i) small interfering RNA in lipid-like nanoparticles resolves liver fibrosis. Hepatology. 2015;62(4):1285–1297. doi: 10.1002/hep.27936. 26096209 PMC4589454

[155] Sato Y, Murase K, Kato J, et al. Resolution of liver cirrhosis using vitamin a-coupled liposomes to deliver sirna against a collagen-specific chaperone. Nat Biotechnol. 2008;26(4):431–442. doi: 10.1038/nbt1396. 18376398

[156] Song N, Xu H, Liu J, et al. Design of a highly potent glp-1r and gcgr dual-agonist for recovering hepatic fibrosis. Acta Pharm Sin B. 2022;12(5):2443–2461. doi: 10.1016/j.apsb.2021.12.016. 35646543 PMC9136578

[157] Zhang L, Schuppan D. Traditional Chinese medicine (TCM) for fibrotic liver disease: hope and hype. J Hepatol. 2014;61(1):166–168. doi: 10.1016/j.jhep.2014.03.009. 24780816

[158] Chi C, Liu X-Y, Hou F, et al. Herbal compound 861 prevents hepatic fibrosis by inhibiting the tgf-beta1/smad/snon pathway in bile duct-ligated rats. BMC Complement Altern Med. 2018;18(1):52. doi: 10.1186/s12906-018-2119-7. 29402324 PMC5800072

[159] Tian Y-L, Zhu X-y, Yin W-W, et al. [Supplemental Fuzhenghuayu capsule therapy for improving liver fibrosis markers in patients with chronic hepatitis b following unsatisfactory outcome of nucleos(t)ide analogue monotherapy]. Zhonghua Gan Zang Bing Za Zhi. 2013;21(7):514–518. doi: 10.3760/cma.j.issn.1007-3418.2013.07.010. 24074710

[160] Yin SS, Wang BE, Wang TL, et al. [The effect of cpd 861 on chronic hepatitis b related fibrosis and early cirrhosis: a randomized, double blind, placebo controlled clinical trial]. Zhonghua Gan Zang Bing Za Zhi. 2004;12(8):467–470. 15329205

[161] Zhang Y, Yao X. Suppressive effects of Yigankang, a combination of Chinese herbs, on collagen synthesis in hepatic stellate cell. J Ethnopharmacol. 2011;134(3):949–952. doi: 10.1016/j.jep.2011.02.014. 21333725

[162] Gu H, Honglian G, Xu LM, et al. Clinical effect of Fuzheng Huayu tablets combined with entecavir in the treatment of chronic hepatitis B liver fibrosis. J Clin Hepatol. 2021;(12):309–313. https://www.clinical+effect+of+fuzheng+huayu+tablets+combined+with+entecavir+in+the+treatment+of+chronic+hepatitis+b+liver+fibrosis.Pdf.

[163] Zhao Z-M, Zhu C-W, Huang J-Q, et al. Efficacy and safety of Fuzheng Huayu tablet on persistent advanced liver fibrosis following 2 years entecavir treatment: a single arm clinical objective performance criteria trial. J Ethnopharmacol. 2022;298:115599. doi: 10.1016/j.jep.2022.115599. 35932973

[164] Qu J, Yu Z, Li Q, et al. Blocking and reversing hepatic fibrosis in patients with chronic hepatitis b treated by traditional Chinese medicine (tablets of Biejia Ruangan or rgt): study protocol for a randomized controlled trial. Trials. 2014;15(1):438. doi: 10.1186/1745-6215-15-438. 25381721 PMC4234899

[165] Wang H, Yang LM, Huang L. [Clinical effects of Qianggan capsule on the liver tissue pathology and pdgf-bb, tgf-beta1, timp-1, and mmp-1 factors in patients with chronic hepatitis B]. Zhongguo Zhong Xi Yi Jie He Za Zhi. 2011;31(10):1337–1340. doi: 10.1007/bf02934426. 22097200

[166] Gu S, Huang MX. [Clinical observation of Qianggan capsule in the treatment of non-alcoholic of non-alcoholic fatty liver fibrosis]. Zhonghua Gan Zang Bing Za Zhi. 2011;19(10):791–792. 22423369

[167] Ferenci P. Silymarin in the treatment of liver diseases: what is the clinical evidence? Clin Liver Dis (Hoboken). 2016;7(1):8–10. doi: 10.1002/cld.522. 31041017 PMC6490246

[168] Clichici S, Olteanu D, Nagy A-L, et al. Silymarin inhibits the progression of fibrosis in the early stages of liver injury in ccl(4)-treated rats. J Med Food. 2015;18(3):290–298. doi: 10.1089/jmf.2013.0179. 25133972

[169] Tsai JH, Liu JY, Wu TT, et al. Effects of silymarin on the resolution of liver fibrosis induced by carbon tetrachloride in rats. J Viral Hepat. 2008;15(7):508–514. doi: 10.1111/j.1365-2893.2008.00971.x. 18397225

[170] Enjalbert F, Rapior S, Nouguier-Soulé J, et al. Treatment of amatoxin poisoning: 20-year retrospective analysis. J Toxicol Clin Toxicol. 2002;40(6):715–757. doi: 10.1081/clt-120014646. 12475187

[171] Sonnenbichler J, Zetl I. Biochemical effects of the flavonolignane silibinin on RNA, protein and DNA synthesis in rat livers. Prog Clin Biol Res. 1986;213:319–331. doi: 10.1016/0006-2952(86)90233-9. 2424029

[172] Jin Y, Wang X, Chen K, et al. Silymarin decreases liver stiffness associated with gut microbiota in patients with metabolic dysfunction-associated steatotic liver disease: a randomized, double-blind, placebo-controlled trial. Lipids Health Dis. 2024;23(1):239. doi: 10.1186/s12944-024-02220-y. 39097726 PMC11297656

[173] Loguercio C, Andreone P, Brisc C, et al. Silybin combined with phosphatidylcholine and vitamin e in patients with nonalcoholic fatty liver disease: a randomized controlled trial. Free Radic Biol Med. 2012;52(9):1658–1665. doi: 10.1016/j.freeradbiomed.2012.02.008. 22343419

[174] Dehmlow C, Erhard J, de Groot H. Inhibition of kupffer cell functions as an explanation for the hepatoprotective properties of silibinin. Hepatology. 1996;23(4):749–754. doi: 10.1053/jhep.1996.v23.pm0008666328. 8666328

[175] Wu JW, Lin LC, Hung SC, et al. Analysis of silibinin in rat plasma and bile for hepatobiliary excretion and oral bioavailability application. J Pharm Biomed Anal. 2007;45(4):635–641. doi: 10.1016/j.jpba.2007.06.026. 17692492

[176] Zarrelli A, Romanucci V, Tuccillo C, et al. New silibinin glyco-conjugates: synthesis and evaluation of antioxidant properties. Bioorg Med Chem Lett. 2014;24(22):5147–5149. doi: 10.1016/j.bmcl.2014.10.023. 25442301

[177] Liu Z-J, Zhong J, Zhang M, et al. The alexipharmic mechanisms of five licorice ingredients involved in cyp450 and nrf2 pathways in paraquat-induced mice acute lung injury. Oxid Med Cell Longev. 2019;2019:7283104–7283120. doi: 10.1155/2019/7283104. 31182998 PMC6512064

[178] Qu Y, Zong L, Xu M, et al. Effects of 18alpha-glycyrrhizin on tgf-beta1/smad signaling pathway in rats with carbon tetrachloride-induced liver fibrosis. Int J Clin Exp Pathol. 2015;8(2):1292–1301. doi: 10.1007/s11596-018-1871-8. 25973013 PMC4396252

[179] Yan T, Wang H, Cao L, et al. Glycyrrhizin alleviates nonalcoholic steatohepatitis via modulating bile acids and meta-inflammation. Drug Metab Dispos. 2018;46(9):1310–1319. doi: 10.1124/dmd.118.082008. 29959134 PMC6081736

[180] Baur JA, Pearson KJ, Price NL, et al. Resveratrol improves health and survival of mice on a high-calorie diet. Nature. 2006;444(7117):337–342. doi: 10.1038/nature05354. 17086191 PMC4990206

[181] Lagouge M, Argmann C, Gerhart-Hines Z, et al. Resveratrol improves mitochondrial function and protects against metabolic disease by activating sirt1 and pgc-1alpha. Cell. 2006;127(6):1109–1122. doi: 10.1016/j.cell.2006.11.013. 17112576

[182] Price NL, Gomes AP, Ling AJY, et al. Sirt1 is required for ampk activation and the beneficial effects of resveratrol on mitochondrial function. Cell Metab. 2012;15(5):675–690. doi: 10.1016/j.cmet.2012.04.003. 22560220 PMC3545644

[183] Aykac M, Balkan E, Gedi Kli S, et al. Resveratrol treatment ameliorates hepatic damage via the tgf-beta/smad signaling pathway in a phenobarbital/ccl(4)-induced hepatic fibrosis model. Iran J Basic Med Sci. 2024;27(9):1124–1133. doi: 10.22038/IJBMS.2024.75737.16398. 39055873 PMC11266736

[184] Kessoku T, Imajo K, Honda Y, et al. Resveratrol ameliorates fibrosis and inflammation in a mouse model of nonalcoholic steatohepatitis. Sci Rep. 2016;6(1):22251. doi: 10.1038/srep22251. 26911834 PMC4766502

[185] Ma Z, Sheng L, Li J, et al. Resveratrol alleviates hepatic fibrosis in associated with decreased endoplasmic reticulum stress-mediated apoptosis and inflammation. Inflammation. 2022;45(2):812–823. doi: 10.1007/s10753-021-01586-w. 35080697 PMC8956545

[186] Faghihzadeh F, Adibi P, Rafiei R, et al. Resveratrol supplementation improves inflammatory biomarkers in patients with nonalcoholic fatty liver disease. Nutr Res. 2014;34(10):837–843. doi: 10.1016/j.nutres.2014.09.005. 25311610

[187] Tu CT, Han B, Liu HC, et al. Curcumin protects mice against concanavalin a-induced hepatitis by inhibiting intrahepatic intercellular adhesion molecule-1 (icam-1) and cxcl10 expression. Mol Cell Biochem. 2011;358(1–2):53–60. doi: 10.1007/s11010-011-0920-4. 21695461

[188] Kong D, Zhang Z, Chen L, et al. Curcumin blunts epithelial-mesenchymal transition of hepatocytes to alleviate hepatic fibrosis through regulating oxidative stress and autophagy. Redox Biol. 2025;84:103690. doi: 10.1016/j.redox.2020.101600. 40467433 PMC12207067

[189] Li B, Wang L, Lu Q, et al. Liver injury attenuation by curcumin in a rat nash model: an nrf2 activation-mediated effect? Ir J Med Sci. 2016;185(1):93–100. doi: 10.1007/s11845-014-1226-9. 25385666

[190] Vizzutti F, Provenzano A, Galastri S, et al. Curcumin limits the fibrogenic evolution of experimental steatohepatitis. Lab Invest. 2010;90(1):104–115. doi: 10.1038/labinvest.2009.112. 19901911

[191] Huang Y, Cao S, Zhang Q, et al. Biological and pharmacological effects of hexahydrocurcumin, a metabolite of curcumin. Arch Biochem Biophys. 2018;646:31–37. doi: 10.1016/j.abb.2018.03.030. 29596797

[192] Wang J, Yu X, Zhang L, et al. The pharmacokinetics and tissue distribution of curcumin and its metabolites in mice. Biomed Chromatogr. 2018;32(9):e4267. doi: 10.1002/bmc.4267. 29689635

[193] Ireson C, et al. Characterization of metabolites of the chemopreventive agent curcumin in human and rat hepatocytes and in the rat *in vivo*, and evaluation of their ability to inhibit phorbol ester-induced prostaglandin e2 production. Cancer Res. 2001;61(3):1058–1064. 11221833

[194] Panahi Y, Kianpour P, Mohtashami R, et al. Efficacy and safety of phytosomal curcumin in non-alcoholic fatty liver disease: a randomized controlled trial. Drug Res (Stuttg). 2017;67(4):244–251. doi: 10.1055/s-0043-100019. 28158893

[195] Saadati S, Sadeghi A, Mansour A, et al. Curcumin and inflammation in non-alcoholic fatty liver disease: a randomized, placebo controlled clinical trial. BMC Gastroenterol. 2019;19(1):133. doi: 10.1186/s12876-019-1055-4. 31345163 PMC6659284

[196] Molokanova O, Schönig K, Weng S-Y, et al. Inducible knockdown of procollagen i protects mice from liver fibrosis and leads to dysregulated matrix genes and attenuated inflammation. Matrix Biol. 2018;66:34–49. doi: 10.1016/j.matbio.2017.11.002. 29122677

[197] Leber N, Kaps L, Aslam M, et al. Sirna-mediated *in vivo* gene knockdown by acid-degradable cationic nanohydrogel particles. J Control Release. 2017;248:10–23. doi: 10.1016/j.jconrel.2016.12.006. 27940184

[198] Melgar-Lesmes P, Luquero A, Parra-Robert M, et al. Graphene-dendrimer nanostars for targeted macrophage overexpression of metalloproteinase 9 and hepatic fibrosis precision therapy. Nano Lett. 2018;18(9):5839–5845. doi: 10.1021/acs.nanolett.8b02498. 30096241 PMC6377857

[199] Ahmed MAA, Kern M, Mourshed B, et al. Fracture resistance of maxillary premolars restored with different endocrown designs and materials after artificial ageing. J Prosthodont Res. 2022;66(1):141–150. doi: 10.2186/jpr.JPR_D_20_00082. 34108294

[200] Neugebauer G, Gabor M, Reiff K. Disposition of carvedilol enantiomers in patients with liver cirrhosis: evidence for disappearance of stereoselective first-pass extraction. J Cardiovasc Pharmacol. 1992;19(Supplement 1):142–146. doi: 10.1097/00005344-199219001-00028. 1378143

[201] El-Demerdash E, Abdel-Sattar SA, El-Bakly WM, et al. Antifibrotic effects of carvedilol and impact of liver fibrosis on carvedilol pharmacokinetics in a rat model. Eur J Drug Metab Pharmacokinet. 2017;42(5):767–779. doi: 10.1007/s13318-016-0391-9. 28012025

[202] Fang H, Yang Y. Designing and validating a robust adaptive neuromodulation algorithm for closed-loop control of brain states. J Neural Eng. 2022;19(3):036018. doi: 10.1088/1741-2552/ac7005. 35576912

[203] Son Y, Kim SJ, Kim HY, et al. Mdm1 ablation results in retinal degeneration by specific intraflagellar transport defects of photoreceptor cells. Cell Death Dis. 2022;13(9):833. doi: 10.1038/s41419-022-05237-2. 36171205 PMC9519634

[204] Tang JW, Toovey OTR, Harvey KN, et al. Introduction of the South African SARS–COV-2 variant 501y.V2 into the UK. J Infect. 2021;82(4):e8–e10. doi: 10.1016/j.jinf.2021.01.007. PMC7813514 33472093

[205] Rahmani S, Asgary S, Askari G, et al. Treatment of non-alcoholic fatty liver disease with curcumin: a randomized placebo-controlled trial. Phytother Res. 2016;30(9):1540–1548. doi: 10.1002/ptr.5659. 27270872

